# Utilizing Electroplex Emission to Achieve External Quantum Efficiency up to 18.1% in Nondoped Blue OLED

**DOI:** 10.34133/2020/8649102

**Published:** 2020-02-27

**Authors:** Xuejun Zhan, Zhongbin Wu, Yanbin Gong, Jin Tu, Yujun Xie, Qian Peng, Dongge Ma, Qianqian Li, Zhen Li

**Affiliations:** ^1^Department of Chemistry, Sauvage Center for Molecular Sciences, Wuhan University, Wuhan 430072, China; ^2^Changchun Institute of Applied Chemistry, The Chinese Academy of Sciences, Changchun 130022, China; ^3^Institute of Molecular Aggregation Science, Tianjin University, Tianjin 300072, China; ^4^Institute of Chemistry, The Chinese Academy of Sciences, Beijing 100190, China; ^5^State Key Laboratory of Luminescent Materials and Devices, South China University of Technology, Guangzhou 510640, China

## Abstract

For the first time, electroplex emission is utilized to enhance the performance of nondoped blue organic light-emitting diodes (OLEDs). By decorating the twisted blue-emitting platform and adjusting the electronic structure, three molecules of 3Cz-Ph-CN, 3Cz-mPh-CN, and 3Ph-Cz-CN with a donor-acceptor structure are synthesized and investigated. When external voltage is applied, electroplex emission, which contributes to the emission performance of OLED, can be realized at the interface between the emitting layer and the electron-transporting layer. Accordingly, high external quantum efficiency of 18.1% can be achieved, while the emission wavelength of the device can be controlled in the blue region. Our results provide the possibility to enhance the performance of OLED through electroplex emission, in addition to the generally investigated thermally activated delayed fluorescence (TADF). Excitedly, when 3Ph-Cz-CN is used as host material in orange-emitting phosphorous OLEDs (PO-01 as the dopant), unprecedented high external quantum efficiency of 27.4% can also be achieved.

## 1. Introduction

Since its discovery in the last century, thanks to unremitting efforts of scientists, organic electroluminescence has witnessed great progress [1–7]. Up to date, organic light-emitting diodes (OLEDs) could even be partially commercialized in full-color flat panel displays and solid-state lighting sources. However, to make full use of the formed excitons and reduce losing energy, it is still essential to seek excellent materials for OLEDs with enhanced light-emitting performances [8–10]. For typical nondoped fluorescent OLEDs, only the singlet (S_1_) excitons corresponding to about 25% of the total electrogenerated excitons could be used [11, 12]. The resultant low external quantum efficiency inspired the utilization of phosphorous OLEDs, which employ transition metal (such as Os, Pt, and Ir) complexes as the emitter [13]. While heavy-atom-induced spin-orbit coupling could make it possible for effectively harvesting triplet excitons (75% of the total electrogenerated excitons) [14], accompanied with higher external quantum efficiencies, the notorious stability problem and difficulty to tune the phosphorous emission of phosphorous OLEDs (PhOLEDs) bothered their wide applications. Besides triplet-triplet annihilation (TTA) [15, 16], pioneered by Uoyama et al., thermally activated delayed fluorescence- (TADF-) based OLEDs have been proposed to harvest both singlet and triplet excitons [17]. The theoretical possibility to fully (100%) make use of the electron-generated excitons was intriguing, and lots of high efficiencies have been reported. However, TADF materials with blue emission were still scarce. The red-shifted emission and stability of the TADF-based OLEDs still need to be further improved though several encouraging examples have been reported [18–20]. Alternatively, other approaches which could efficiently make use of the excitons formed inside the OLED devices still needed to be explored.

**Scheme 1:**
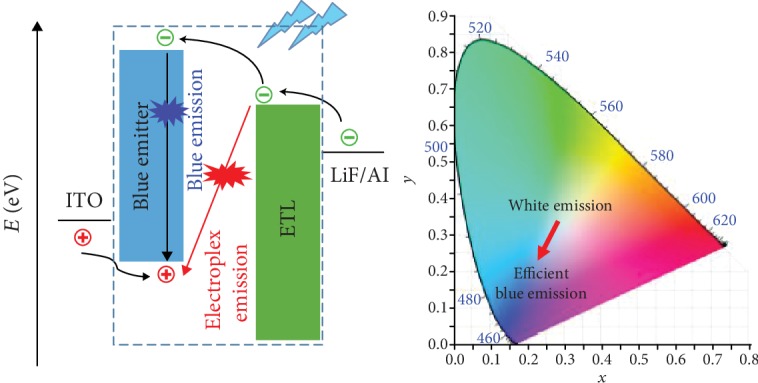
Schematic description of the route to utilize exciplex/electroplex emission in blue OLED.

Considering the above molecular designs carefully, all the attempts are utilizing the intramolecular excitons. Actually, taking TADF as an example, the excited states with essential small Δ*E*_ST_ can also be achieved by exciplex excitons, which can be generated by two oppositely charged molecules with weak Coulomb interactions [21]. Early in 1999, reported by Shirota et al., electron-transporting material of 5,5′-bis(dimesitylboryl)-2,2′-bithiophene (BMB-2T) was found to form an exciplex with the hole-transporting material of TPD at the interface [22]. After that, the EL efficiency and stability of bilayer-type exciplex OLED could be improved stepwise [23–25], indicating that it was possible to utilize intermolecularly formed excitons for the enhancement of the OLED performance. Actually, electroplex [26], similar to exciplex, was also a transient donor-acceptor complex between the excited state of the donor and the ground state of the acceptor. But different from exciplex, the newly formed peaks in EL spectra caused by the electroplex emission could not be observed in the PL spectra of the blend films of donor and acceptor molecules. Generally, the newly formed red-shifted broad bands were employed to realize white emission [27, 28], and there were no efforts of using electroplex or exciplex to enhance the device performance while controlling the red-shifted bands. In recent years, our group has focused on the research of blue OLEDs with the emphasis on the design of blue and deep-blue AIEgens ([Supplementary-material supplementary-material-1]) [29–31]; accordingly, we wondered that if we choose a deep-blue emitter with the properly controllable red-shifted bands, the overall blue emission OLED with high performance should be possible by utilizing the formation of electroplex or exciplex (Scheme 1).

Twisted architecture has been proved to be effective for the realization of deep-blue emission [31]; thus, the easily modified 2,4,6-tribromobenzonitrile unit was chosen as the construction core. Carbazole, a good hole-transporting moiety which is easy to generate electroplex/exciplex [27], was employed to be the periphery substitution. To subtly adjust the distribution of electrons, here, three carbazole-based luminogens of 3Cz-Ph-CN, 3Cz-mPh-CN, and 3Ph-Cz-CN were synthesized with different linkage models. Excitedly, electroplex could be formed at the interface between the emitting layer (EML) and the electron-transporting layer (ETL), when 3Ph-Cz-CN was used as EML in nondoped OLEDs. As expected, the high external quantum efficiency of 18.1% could be achieved, while the emission wavelength of the device could be controlled in the blue region. To the best of our knowledge, this is the first time that electroplex has been successfully utilized to enhance the performance of nondoped blue OLEDs. Furthermore, by utilizing 3Ph-Cz-CN as host material in orange-emitting PhOLEDs (PO-01 as the dopant), unprecedented high external quantum efficiency of 27.4% could be realized. Herein, we would like to present the synthesis and thermal, photophysical, electrochemical, and electroluminescent properties in detail.

## 2. Results

### 2.1. Synthesis and Characterization

**Figure 1:**
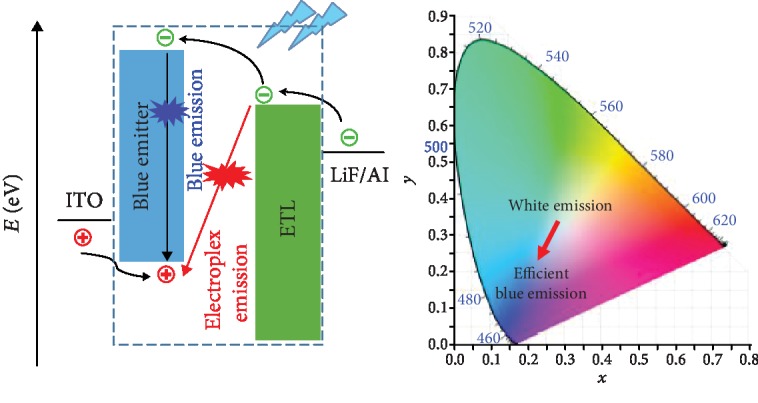
Chemical structures, molecular orbital amplitude plots, and theoretical energy levels of (a) 3Cz-Ph-CN, (b) 3Cz-mPh-CN, and (c) 3Ph-Cz-CN calculated by B3LYP/6-31G (d, p).

Through cyanation and the followed Suzuki coupling reactions, all the cyano-containing luminogens (Figure 1) were easily synthesized and purified by column chromatography with fine to good yields ([Supplementary-material supplementary-material-1]). Compared to 3Cz-Ph-CN, due to the larger intramolecular twisting degree [29], 3Cz-mPh-CN and 3Ph-Cz-CN exhibited better solubility. Their chemical structures were definitely characterized by ^1^H and ^13^C NMR, mass spectrometry, and elemental analysis. Thermal properties of the new luminogens were investigated by thermogravimetric analysis (TGA) and differential scanning calorimetry (DSC) measurements ([Supplementary-material supplementary-material-1]). As expected, owing to the good thermal stability of carbazole units, all the carbazole-containing luminogens were thermally stable with high *T*_d_ (corresponding to 5% weight loss) values surpassing 500°C. Generally, because of the more rigid conformations, compounds with *para*-linkage mode show better thermal stability than those with *meta*-linkage way [29]. Thus, among them, 3Cz-Ph-CN exhibited excellent thermal stability with the *T*_d_ value of 550°C, much higher than that of 3Cz-mPh-CN (504°C) with the *meta*-linkage mode. The glass transition temperatures (*T*_g_) for 3Cz-Ph-CN, 3Cz-mPh-CN, and 3Ph-Cz-CN were 165, 121, and 141°C, respectively. These high *T*_g_ values could guarantee proper operating stability of the fabricated OLED devices [8].

### 2.2. Optical Properties

**Figure 2:**
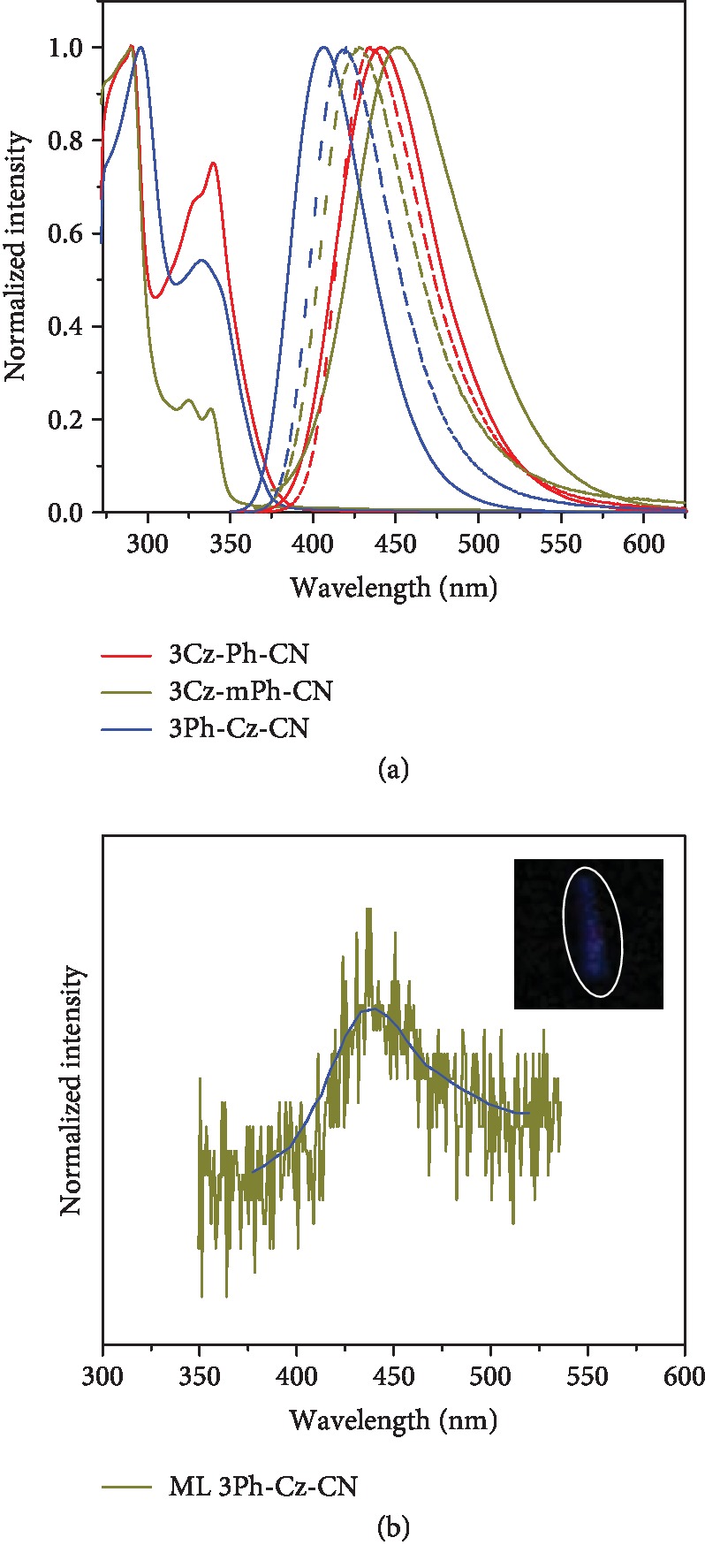
(a) UV-vis spectra and PL spectra of 3Cz-Ph-CN, 3Cz-mPh-CN, and 3Ph-Cz-CN in THF solutions (10 *μ*M, dotted lines represent the PL spectra of films). (b) Mechanoluminescence spectrum of 3Ph-Cz-CN (inset is the ML image in the dark).

Absorption spectra of the carbazole-based luminogens in dilute THF solutions showed two bands located at 290 and 340 nm (Figure 2(a)), which could be ascribed to the carbazole-centered *n*‐*π*^∗^ transition and intramolecular charge transfer (ICT) transition, respectively. In UV-vis spectra, 3Cz-Ph-CN showed red-shifted absorption compared to that of 3Cz-mPh-CN or 3Ph-Cz-CN, indicating its relatively larger conjugation. For 3Cz-mPh-CN and 3Ph-Cz-CN, the absorption onset values were 356 and 382 nm, respectively. The blue-shifted absorption onset of 3Cz-mPh-CN indicated that the molecule adopting the *meta*-connection could induce more twisted architecture. Besides, the peak intensity of the bands aroused by ICT transition could also give some information about the intramolecular charge transfer effect. In 3Cz-Ph-CN, due to its better conjugation between the periphery carbazole units and the center benzene unit, the charge transfer effect was more obvious than those of 3Cz-mPh-CN and 3Ph-Cz-CN.

In solutions, all the luminogens exhibited fluorescence with blue emission. Similar to our previous results [32], the changing of the connection mode from *para*-linkage to *meta*-linkage did not alter the emission largely. As shown in Figure 2(a) and Table 1, the emission wavelength of 3Cz-Ph-CN is located at 441 nm. By adopting the *meta*-linkage way, regardless of the reduced conjugation, 3Cz-mPh-CN showed slightly red-shifted emission. However, 3Ph-Cz-CN with carbazole units connected directly at the benzene core showed the obvious blue-shifted emission compared with those of 3Cz-Ph-CN and 3Cz-mPh-CN. This indicates that the connection through 3-position of the carbazole unit was effective to achieve deep-blue emission in this case.

Typically, for organic molecules, *π*‐*π* stacking in the film state will make the emission red-shifted. However, as shown in Table 1, the fluorescent spectrum of 3Cz-mPh-CN showed obvious blue-shifted emission compared with that in solution [32]. To understand this result, two factors should be taken into consideration. In the solution state, the polarity of solvent could enhance the intramolecular charge transfer (ICT) effect, which will make the emission red-shifted. However, in the film state, effect caused by polarity of solvent disappeared. This makes the blue-shifted emission reasonable. Another factor that needs to be discussed was the twisted architecture. The highly twisted structure of the molecule inhibited the *π*‐*π* stacking in the film state. In other words, the red-shifted emission phenomenon will be less obvious for the molecule with the highly twisted structure. What we observed should be an overall result caused by these two factors.

**Figure 3:**
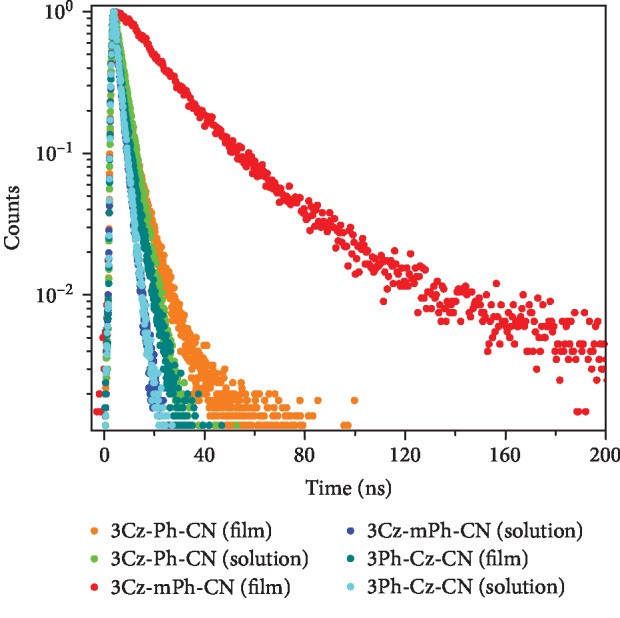
Emission decay characteristics of the three luminogens (film and solution states) at room temperature.

In the fluorescent spectra of the films, 3Cz-mPh-CN showed slightly blue-shifted emission (428 nm) compared with that of 3Cz-Ph-CN (435 nm). No matter the red-shifted emission compared to its solution state, 3Ph-Cz-CN could show deep-blue emission (420 nm) with narrow fwhm (full width at half-maximum) of 56 nm, which was beneficial for the realization of stable deep-blue emission in OLED devices. In order to quantitatively know the fluorescent properties of these new luminogens, absolute fluorescent quantum yields and fluorescent lifetimes were measured. Benefiting from the highly emissive intramolecular charge transfer (ICT) excited states [20], 3Cz-Ph-CN and 3Ph-Cz-CN showed high fluorescent quantum yields (Table 1). The fluorescent quantum yields of 3Cz-Ph-CN, 3Cz-mPh-CN, and 3Ph-Cz-CN were 90.1%, 7.7%, and 65.9%, respectively. With twisted structures and ICT effects inside the luminogens, the possible TADF emission was studied. As shown in Figure 3, all the luminogens exhibited first-order exponential decays with a short fluorescent lifetime (2.35-3.88 ns) in THF solutions, indicating that no thermally activated delayed fluorescence (TADF) emission existed. The absence of TADF emission could also be verified by the energy gap between the lowest singlet state and the lowest triplet state (Δ*E*_st_). From the low-temperature fluorescent and phosphorescent spectra ([Supplementary-material supplementary-material-1]), the Δ*E*_st_ values of 3Cz-Ph-CN, 3Cz-mPh-CN, and 3Ph-Cz-CN were calculated to be 0.46, 0.50, and 0.56 eV, respectively. According to Adachi's results [17], these values were not small enough to guarantee the efficient reverse intersystem crossing. These results showed that no TADF emission was present to contribute to the intense fluorescent emission and electroluminescent emission in the following parts.

Mechanoluminescence (ML), emission caused by the mechanical force on powders/crystals, has attracted intense interests in recent years [33, 34]. It was interesting that 3Ph-Cz-CN was ML active. As shown in Figure 2(b), by scratching the powders, deep-blue emission could be observed by naked eyes without irradiation with a UV lamp. According to recent literature results, this unexpected ML property possibly originated from the strong intermolecular interactions [35]. However, regardless of many efforts, the crystal of 3Ph-Cz-CN could not be obtained for the detailed research. However, it is still to be noted that this is the first example of an organic molecule with molecular weight up to 826 g/mol possessing the ML property [36]. Also, the previous ML examples exhibited sky-blue to red ML emission, and the cyano-containing ML molecule with a deep-blue ML spectrum (*λ*_em_ = 438 nm) has never been reported. Thus, our case could provide valuable information for the study of ML molecules, and further investigation was required.

### 2.3. Energy Levels and Theoretic Calculations

To get information about the energy levels, cyclic voltammetry (CV) measurements were carried out ([Supplementary-material supplementary-material-1]). As the hole mobilities of the carbazole-based luminogens were higher than electron mobilities, the highest occupied molecular orbital (HOMO) energy levels of the luminogens were estimated from the onset oxidation potentials according to the equation of HOMO = −(4.80 + *E*_ox_) eV, while the lowest unoccupied molecular orbital (LUMO) energy levels were calculated by optical band gap energies and HOMO values [30]. As shown in Table 1, the HOMO energy levels for 3Cz-Ph-CN, 3Cz-mPh-CN, and 3Ph-Cz-CN are similarly located at around -5.60 eV. Since the HOMO energy level of NPB, the traditional hole-transporting layer material, is -5.30 eV, these new luminogens could act as light-emitting layers in OLED devices. Besides, their corresponding LUMO energy levels were calculated to be -2.38, -2.10, and -2.37 eV, respectively. As the LUMO level of traditional electron-transporting material TPBi is about -2.7 eV, the transfer of electrons would be easy from the TPBi layer to the light-emitting layer in 3Cz-Ph-CN/3Ph-Cz-CN-based OLED devices. Moreover, the large band gap of 3Cz-Ph-CN (3.48 eV) compared to other carbazole-based emitters indicated its poor conjugation and weak ICT effect, owing to the more twisted structure [8, 32].

Calculated by B3LYP/6-31G (d, p), molecular orbital amplitude plots and theoretical energy levels of the three luminogens were shown in Figure 1. Like other molecules with electron-donors and electron-acceptors, electron clouds in the HOMOs are mainly located on the carbazole units while those in LUMOs were mainly at the center benzene units. In the three luminogens, there were slight overlaps between the HOMOs and LUMOs, which could be beneficial for the transfer of both electrons and holes when applied in OLED devices [8, 31]. In the optimized structures, twist angles between the substitution units (*ortho*-position of the cyano group) and the central benzene were larger than those between the substitution units (*para*-position of the cyano group) and the central benzene, indicating that the cyano group benefited to the twisting conformation inside luminogens. Actually, the cyano groups were critical for the inhibition of the aggregation-induced quenching (ACQ) effect and the increase of the quantum yields in solid states. Besides, the twist angles between the cyano group and the *ortho*-substitution units were 49.03 , 52.74 , and 51.41 for 3Cz-Ph-CN, 3Cz-mPh-CN, and 3Ph-Cz-CN, respectively, indicating that the construction of molecules through the *meta*-linkage approach was an efficient method to make molecules twist.

### 2.4. Characterization of Nondoped Devices

**Figure 4:**
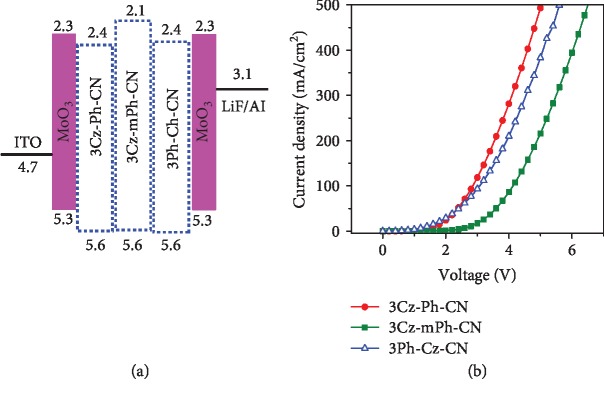
(a) Device structures and (b) current density-voltage profiles of hole-only devices based on the three luminogens.

Prior to the fabrication of OLED devices, hole-only devices with the typical configuration of ITO/MoO_3_ (10 nm)/X (15 nm)/MoO_3_ (10 nm)/Al were fabricated to evaluate the hole-transporting ability of the carbazole-based luminogens, in which MoO_3_ layers functioned as both hole-injection and electron-blocking layers and X referred to the newly synthesized luminogens of 3Cz-Ph-CN, 3Cz-mPh-CN, or 3Ph-Cz-CN. As shown in Figure 4, the current density-voltage (*J*‐*V*) profiles indicated that all the three new luminogens exhibited good hole-transport ability [37]. Among them, 3Cz-Ph-CN with the *para*-linkage mode demonstrated superior hole-transport ability. Even though 3Cz-mPh-CN and 3Ph-Cz-CN both possessed twisted architecture, the better transport ability of 3Ph-Cz-CN indicated that the connection through 3-position of the carbazole unit was beneficial for achieving better transport ability while maintaining the twisted structure.

**Figure 5:**
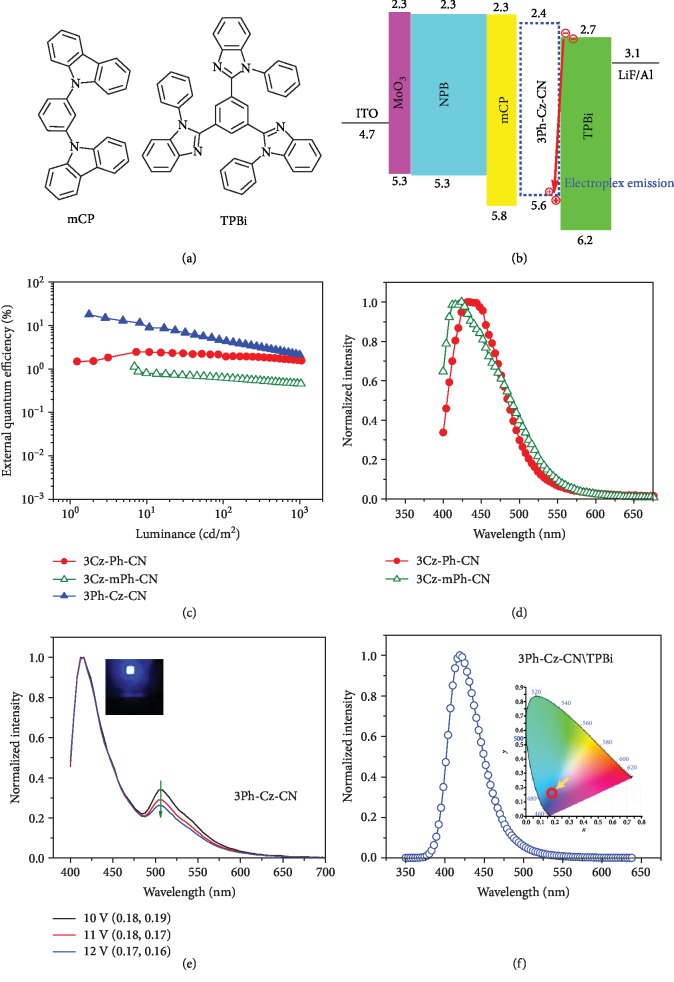
(a) Chemical structures of mCP and TPBi. (b) Energy level of nondoped devices with electroplex emission. Device configurations: ITO/MoO_3_ (10 nm)/NPB (60 nm)/mCP (15 nm)/EML (30 nm)/TPBi (30 nm)/LiF (1 nm)/Al. (c) External quantum efficiency-luminance characteristics of the nondoped devices. (d) EL spectra of 3Cz-Ph-CN and 3Cz-mPh-CN. (e) EL spectra of 3Ph-Cz-CN at different voltages (inset is the picture of device C at the voltage of 12 V). (f) PL spectrum of the blend film (inset is the description of CIE in the nondoped device C).

After the survey of hole-only devices, nondoped fluorescent OLED devices were fabricated with the configuration of ITO/MoO_3_ (10 nm)/NPB (60 nm)/mCP (15 nm)/X (30 nm)/TPBi (30 nm)/LiF (1 nm)/Al (Figure 5), in which MoO_3_, NPB, and TPBi served as hole-injection, hole-transporting, and electron-transporting layers. Due to the high triplet state energy (*E*_T_), the applied neat film of mCP had been proved to have the ability of confining excitons in the emitting layer (EML) [8]. As shown in Figure 5 and Table 2, all the fabricated devices exhibited blue to deep-blue emission as expected. For 3Cz-Ph-CN and 3Cz-mPh-CN, the highest external quantum yields (EQEs) were 2.45% and 1.14%, respectively. The high turn-on voltage (*V*_on_) gave some clues that the device structures could be further optimized. For the 3Ph-Cz-CN-based devices, it was quite shocking that the EQE value could be as high as 18.1%.

Generally, the theoretically determined maximum quantum yield for fluorescent OLEDs could be expressed by the following equation [38, 39]:

(1) $$\begin{array}{c} \eta_{\text{ext}}=\eta_{\mathrm{int}}\times \eta_{\text{out}}=\left(\gamma \times \eta_{\text{ST}}\times \Phi_{\text{PL}}\right)\times \eta_{\text{out}}, \end{array}$$

where the external quantum efficiency (*η*_ext_) is determined by the internal quantum efficiency (*η*_int_) and light outcoupling factor (*η*_out_, typically 0.2-0.3). *η*_int_ is determined by the charge balance factor (*γ*, ideally 1.0 when carriers inside the device were fully balanced), fraction of radiative excitons (*η*_ST_, 0.25 for typical fluorescent emitters) and PL quantum yield of the emitting layer (*Φ*_PL_). In our case, as the value of *Φ*_PL_ (film state) was determined to be 41.4%, the theoretical calculated external quantum efficiency should be 2.1-3.1%. However, the actual external quantum efficiency was obviously higher (6-9 times) than the calculated one, unambiguously indicating that some other processes were involved.

With the low *V*_on_ value (2.8 V), the device demonstrated excellent efficiencies with the maximum power efficiency (PE) of 24.9 lm W^−1^ and current efficiency (CE) of 23.8 cd A^−1^. According to the emission data summarized in Table 1, 3Ph-Cz-CN with the highly twisted structure should demonstrate a blue-shifted emission compared to other luminogens. However, due to the shoulder peak located at 506 nm (Figure 5(e)), devices based on 3Ph-Cz-CN showed larger CIE_*y*_ values. Also, along with slightly blue-shifted characteristics, the intensity of the shoulder peak would decrease when higher voltages were applied. At the voltage of 10 V, the device showed CIE of (0.18, 0.19). However, the CIE of (0.17, 0.16) was observed when the applied voltage was increased to be 12 V. This made it possible for making the emission color varied through subtly changing the applied voltage. And we suspected that the unprecedented high EQE value should have close relationship with the newly formed shoulder peak in the EL spectrum.

According to the previous results reported in literatures [22, 28, 40], the newly formed peaks in EL spectra were possibly related to phosphorous emission, exciplex emission, or/and electroplex emission. As the shoulder peak could not be observed in the PL spectrum of the solid film, it should not be caused by phosphorous emission [40]. To get further understanding of the above phenomenon, we tested the fluorescent spectrum of the blend film of 3Ph-Cz-CN and TPBi. As shown in Figure 5(f), the blend film also exhibited an emission peak similar to the neat film of 3Ph-Cz-CN. Then, the shoulder peak should not be caused by the reported phenomenon of exciplex emission. Actually, the observed phenomena of increased CIE_*y*_ and voltage-related EL spectra were the characteristics of electroplex emission [27]. According to Kalinowski et al.'s model [41], at the interface of 3Ph-Cz-CN (D) and TPBi (A), (D^+^) and (A^−^) could form two possible configurations: locally excited (LOC) configuration |A∗D〉 and charge transfer (CT) configuration |A^−^D^+^〉. The coefficients of *C*_1_ and *C*_2_ determine the extent of mixing between LOC and CT configurations:

(2) $$\begin{array}{c} \Psi_{\text{EX}}=C_{1}|\text{A}*\text{D}\rangle +C_{2}|{\text{A}}^{‐}{\text{D}}^{+}\rangle . \end{array}$$

For a special donor and acceptor, when the gap between LUMO of the acceptor and LUMO of the donor is large, electrons at LUMO of the acceptor can directly cross recombine with the holes at the HOMO of the donor. This procedure produces the CT configuration, accompanied with the electroplex emission [42]. In Song et al.'s case [28], the LUMO gap between 3,3-di(9H-carbazol-9-yl)biphenyl (mCBP) and 2,8-bis(4,6-diphenyl-1,3,5-triazin-2-yl)dibenzo[b,d]furan (DBFTrz) was around 0.5 eV. However, here, for 3Ph-Cz-CN and TPBi, the electron at the LUMO of TPBi could directly cross recombine with the holes at the HOMO of 3Ph-Cz-CN even though the LUMO gap was just 0.3 eV. This case could provide more information for the deep understanding of the formation of electroplex emission. Based on the above analysis, there is no doubt that the CT configuration contributed to the high efficiency of OLED devices. To the best of our knowledge, this is the first example, which achieved unprecedented EQE value up to 18.1% in nondoped blue organic light-emitting diodes by utilizing electroplex emission [28, 29].

To know more about the emissive properties of electroplex emission, we also carried out transient EL experiments. [Supplementary-material supplementary-material-1] showed the EL spectra when different voltages were applied. For the emission located around 410 nm, the transient EL spectra exhibited obvious overshoot which declined when higher voltage was applied. Combining with literature reports [43], the overshoot was caused by built-in voltage created by accumulated carriers. When higher voltage was applied, there were more traps in the emitter. And the carriers accumulated in the interface would easily recombine with the traps. This reduced the effect of build-in voltage, and thus, the overshoot diminished. However, for the electroplex emission around 510 nm, the transient EL spectra were almost identical when different voltages were applied. We anticipated that the electroplex located at the interface of the emitter and EML could be weakly influenced by the traps in the emitter. Comparing the transient spectra at different wavelengths, we could draw the conclusion that the lifetime of electroplex emission was longer than that of the fluorescent emission (410 nm). And when higher voltage was applied, the lifetime of electroplex emission showed an increased trend.

Considering the excellent hole-transporting ability of the three luminogens, we wondered whether the device structures could be simplified by removing the hole-transporting layer (NPB) and exciton-confining layer (mCP) [31]. Then, devices with the configuration of ITO/MoO_3_ (10 nm)/X (60 nm)/TPBi (30 nm)/LiF (1 nm)/Al were fabricated ([Supplementary-material supplementary-material-1]). As shown in [Supplementary-material supplementary-material-1] and Table 2, the CIE and EQE were slightly improved for the devices employing 3Cz-Ph-CN-based devices. But for 3Cz-mPh-CN and 3Ph-Cz-CN, perhaps, the hole-transporting abilities were not high enough for the possibilities of removing hole-transporting layers. Besides, in the 3Ph-Cz-CN-based devices, the electroplex emission could not be observed, demonstrating that the properly designed device structure should also be critical for the utilization of electroplex emission.

### 2.5. Characterization of Doped Devices

**Figure 6:**
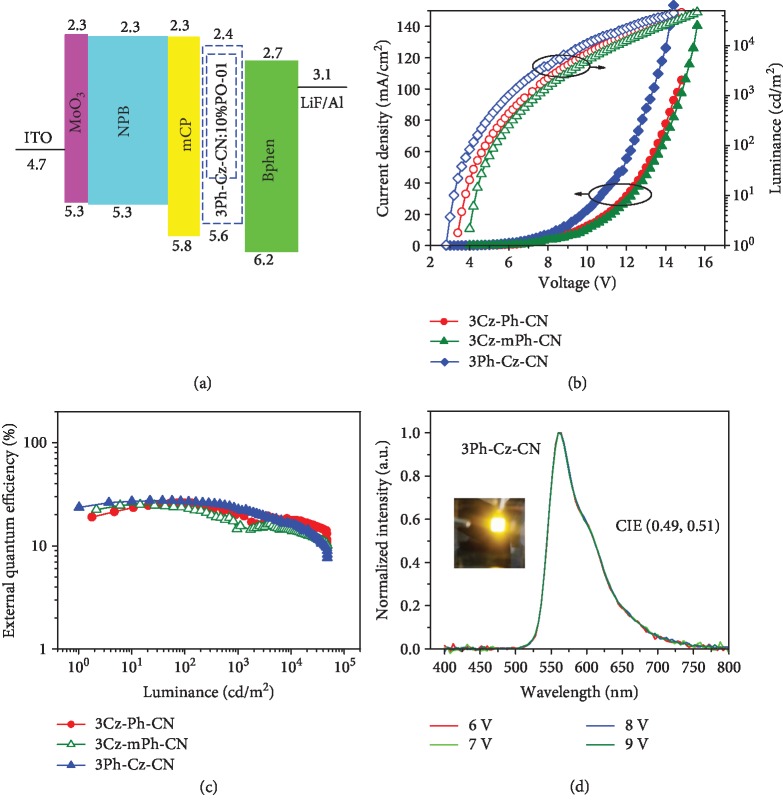
(a) Energy level of doped devices. (b) Current density-voltage-luminance characteristics and (c) change in external quantum efficiency with the luminance in the doped devices. (d) EL spectrum of 3Ph-Cz-CN at different voltages in device I (inset is the picture of device I at the voltage of 8 V). Device configurations: ITO/MoO_3_ (10 nm)/NPB (60 nm)/mCP (10 nm)/X:PO-01 (20 nm, 10 wt%)/Bphen (40 nm)/LiF (1 nm)/Al.

CN-containing luminogens had been reported to be used as efficient host material in OLED devices [31, 44]. Considering the triplet energy level, doped devices with the configuration of ITO/MoO_3_ (10 nm)/NPB (60 nm)/mCP (10 nm)/X:PO-01 (20 nm, 10 wt%)/Bphen (40 nm)/LiF (1 nm)/Al were fabricated, in which Bphen (bathophenanthroline) was used as the electron-transporting layer (Figure 6, devices G-I). All the fabricated devices demonstrated stable orange emission with high efficiencies. For 3Cz-mPh-CN-based doped devices, *V*_on_ and maximum luminance (*L*_max_) was 3.8 V and 46401 cd m^−2^, respectively. The EL spectra were almost unchanged when different voltages were applied. With the EL spectrum located at 564 nm, the external quantum efficiency could be as high as 25.1%. For 3Cz-Ph-CN, *V*_on_ was reduced to be 3.4 V, indicating the improved matching of energy levels [45]. As the result, the device demonstrated improved performance with EQE of 26.1%. It is interesting that for the device I employing 3Ph-Cz-CN as the host material, unprecedented high EQE of 27.4% could be achieved. It was worth noting that this was the highest external quantum efficiency for the PO-01-based phosphorous OLED [23, 31, 44]. For the detailed parameters, *V*_on_ could be decreased to be as low as 2.8 V, indicating the easier transfer of carriers inside the fabricated device. With improved luminance (47952 cd m^−2^), the device exhibited maximum PE and CE of 84.5 cd m^−2^ and 84.6 cd A^−1^, respectively. From the characteristics in Figure 6(c) and Figure 6(d), device I demonstrated excellent stability and low efficiency roll-off. In particular, at the luminance of 1000 cd m^−2^, maximum CE and EQE of the doped device could still remain 70.3 cd m^−2^ and 22.2%, respectively. All the results demonstrated that the easily synthesized luminogens could be an excellent host for orange emission. Benefit from the donor-acceptor structures and good transport abilities, the high efficiencies, and small roll-off indicated that triplet-triplet annihilation and exciton-polaron annihilation inside the devices were efficiently inhibited [46]. However, compared with the other two hosts, the 3Cz-mPh-CN-based device exhibited relatively lower efficiency and higher *V*_on_ value (Table 2). This was mainly attributed to the relatively larger LUMO gap between 3Cz-mPh-CN and Bphen ([Supplementary-material supplementary-material-1]). When utilized as host materials, regardless of similar HOMO/LUMO energy levels and triplet energy levels, 3Ph-Cz-CN with relatively poor hole-transport ability exhibited better device performance than that of 3Cz-Ph-CN. Because of the hole-dominated transport property [18, 29, 42], the reason could be attributed to the more balanced carrier transfer inside the 3Ph-Cz-CN-based device [46]. Another reason could be attributed to the relatively strong intermolecular *π*‐*π* stacking in the 3Cz-Ph-CN films, which was not beneficial for the inhibition of concentration quenching [47]. Adversely, the larger twisting degree inside 3Ph-Cz-CN benefited for the maintenance of the amorphous state.

## 3. Discussion

In summary, luminogens with good stability and robust emission were synthesized through simple procedures. By utilizing the electroplex emission, TADF-absent luminogen of 3Ph-Cz-CN exhibited the unprecedented high external quantum efficiency of 18.1% in nondoped devices, providing a new possibility to greatly enhance the performance for OLEDs with blue emissions. Furthermore, orange-emitting PhOLEDs employing 3Ph-Cz-CN as host material provided the maximum CE and EQE of 84.6 cd A^−1^ and 27.4%, respectively, with small efficiency roll-off. The achieved EQE value is the highest external quantum efficiency for PO-01-based phosphorous OLED up to date. At the luminance of 1000 cd m^−2^, maximum CE and EQE of the doped device can still remain 70.3 cd m^−2^ and 22.2%, respectively, confirming the advantages of 3Ph-Cz-CN as host material for orange-emitting PhOLEDs.

## 4. Materials and Methods

### 4.1. Characterization

^1^H and ^13^C NMR spectra were measured on WNMR-I NMR (600 MHz) and Bruker AVANCE III HD (400 MHz) spectrometers, respectively. Elemental analyses of carbon (C), hydrogen (H), and nitrogen (N) were performed on a CARLO ERBA-1106 microanalyzer. The MS (EI) spectrum was recorded on a Finnigan PRACE mass spectrometer. MALDI-TOF spectra were measured with a Bruker Autoflex III mass spectrometer operating in MALDI-TOF (matrix-assisted laser desorption/ionization-time-of-flight) mode with 1,8,9-anthracenetriol as the matrix. For photophysical properties, UV-vis absorption spectra were recorded on a Shimadzu UV-2500 recording spectrometer while the photoluminescence spectra were recorded on a Hitachi F-4500 fluorescence spectrometer. For low-temperature tests, samples were all cooled in liquid nitrogen. The emission decay profiles were measured with a FLS980 fluorescence lifetime spectrometer. In the atmosphere of nitrogen, thermogravimetric analysis (TGA) and differential scanning calorimetry (DSC) were performed with NETZSCH STA 449C and Mettler Toledo DSC 822e instrument, respectively. Cyclic voltammetry (CV) curves of the three emitters were obtained on a CHI voltammetric analyzer in a three-electrode cell. The three electrodes were the Pt counter electrode, Ag/AgCl reference electrode, and glassy carbon working electrode. The scans were performed at the rate of 100 mV s^−1^ with tetrabutylammonium perchlorate (0.1 M, anhydrous dichloromethane solution purged with nitrogen) as the supporting electrolyte. All the potential values obtained were converted to values versus the saturated calomel electrode (SCE) by using ferrocenium/ferrocene (Fc^+^/Fc) as the internal standard. The electronic and geometrical properties of all the three luminogens were optimized by Gaussian 09 program (B3LYP/6-31 g(d) level).

### 4.2. OLED Device Fabrication and Measurement

Beside light-emitting layers, all the materials were obtained from a commercial source. Before deposition of the organic layer, clean ITO substrates were treated with oxygen plasma for 2 min. EL devices were fabricated by vacuum deposition of the materials at a base pressure of 10^−6^ Torr onto glass with a layer of indium tin oxide (ITO) with a sheet resistance of 25 *Ω*/square. The deposition rate of organic compounds was controlled to be 1-2 Å s^−1^. Finally, a cathode composed of lithium fluoride (LiF, 1 nm) and aluminium (Al, 100 nm) was carefully deposited onto the substrate in the vacuum of 10^−6^ Torr. The *L*‐*V*‐*J* characteristics of the fabricated devices were measured with a Keithley 2400 Source meter and a Keithley 2000 Source multimeter equipped with a calibrated silicon photodiode. EL spectra of devices were measured by a JY SPEX CCD3000 spectrometer. All measurements were carried out at room temperature under ambient conditions.

#### 4.2.1. Synthesis of 3Cz-Ph-CN

A mixture of 2,4,6-tribromobenzonitrile (337 mg, 1.0 mmol), (4-(*9H*-carbozol-9-yl)phenyl)boronic acid (1.43 g, 5.0 mmol), Pd(PPh_3_)_4_ (50 mg) and potassium carbonate (4.14 g, 30 mmol) in toluene (35 mL), EtOH (7.0 mL), and distilled water (15 mL) was refluxed for 24 h under nitrogen in a 250 mL Schlenk tube. After quenching by water, the resultant mixture was extracted with dichloromethane. Then, the combined organic extracts were dried over anhydrous Na_2_SO_4_ and concentrated by rotary evaporation. The crude product was purified by column chromatography on silica gel using dichloromethane/petroleum ether as eluent to afford the product as light yellow solid in the yield of 56% (461 mg). ^1^H NMR (600 MHz, CDCl_3_) *δ* (ppm): 8.19 (d, *J* = 7.8 Hz, 6H), 8.03-7.80 (m, 8H), 7.82 (d, *J* = 7.8 Hz, 4H), 7.79 (d, *J* = 7.8 Hz, 2H), 7.59 (d, *J* = 8.4 Hz, 4H), 7.52-7.46 (m, 8H), 7.35-7.32 (m, 6H). ^13^C NMR (100 MHz, CDCl_3_) *δ* (ppm): 140.6, 138.6, 137.3, 130.7, 129.0, 128.0, 127.7, 127.2, 126.1, 123.6, 120.5, 120.4, 120.3, 109.9, 109.7. MS (MALDI-TOF), m/z: 826.45 ([M^+^], calcd for C_61_H_38_N_4_, 826.31). Anal. Calcd for C_61_H_38_N_4_: C, 88.59; H, 4.63; N, 6.77. Found: C, 88.43; H, 4.85; N, 6.51.

#### 4.2.2. Synthesis of 3Cz-mPh-CN

The synthetic procedure was similar to that of 3Cz-Ph-CN. 2,4,6-Tribromobenzonitrile (200 mg, 0.59 mmol) reacted with (3-(*9H*-carbozol-9-yl)phenyl)boronic acid (852 mg, 2.97 mmol), in the presence of Pd(PPh_3_)_4_ (50 mg) and potassium carbonate (2.48 g, 18.0 mmol), to yield 3Cz-mPh-CN (white solid, 72%, 351 mg). ^1^H NMR (600 MHz, CDCl_3_) *δ* (ppm): 8.15 (d, *J* = 7.8 Hz, 6H), 7.86 (s, 3H), 7.82 (s, 2H), 7.78-7.71 (m, 8H), 7.65 (d, *J* = 7.2 Hz, 1H), 7.59 (d, *J* = 8.4 Hz, 4H), 7.43-7.38 (m, 8H), 7.31-7.28 (m, 6H). ^13^C NMR (100 MHz, CDCl_3_) *δ* (ppm): 146.7, 144.4, 140.8, 140.7, 140.2, 138.7, 138.3, 130.9, 130.4, 128.0, 127.9, 127.8, 127.6, 126.5, 126.1, 123.5, 123.4, 120.5, 120.3, 120.2, 110.0, 109.6. MS (MALDI-TOF), m/z: 826.38 ([M^+^], calcd for C_61_H_38_N_4_, 826.31). Anal. Calcd for C_61_H_38_N_4_: C, 88.59; H, 4.63; N, 6.77. Found: C, 88.53; H, 4.74; N, 6.49.

#### 4.2.3. Synthesis of 3Ph-Cz-CN

The synthetic procedure was similar to that of 3Cz-Ph-CN. 2,4,6-Tribromobenzonitrile (200 mg, 0.59 mmol) reacted with (9-phenyl-*9H*-carbazol-3-yl)boronic acid (852 mg, 2.97 mmol), in the presence of Pd(PPh_3_)_4_ (50 mg) and potassium carbonate (2.48 mg, 18.0 mmol) to yield 3Ph-Cz-CN (white solid, 63%, 307 mg). ^1^H NMR (600 MHz, CDCl_3_) *δ* (ppm): 8.54 (s, 1H), 8.50 (s, 2H), 8.25 (d, *J* = 7.8 Hz, 2H), 8.23 (d, *J* = 7.8 Hz, 1H), 7.97 (s, 2H), 7.84-7.81 (m, 3H), 7.65-7.61 (m, 12H), 7.58 (d, *J* = 9.0 Hz, 2H), 7.52-7.45 (m, 10H), 7.34-7.32 (m, 3H). ^13^C NMR (100 MHz, CDCl_3_) *δ* (ppm): 148.3, 145.7, 141.5, 141.4, 141.1, 141.0, 137.5, 137.4, 131.4, 131.1, 130.1, 130.0, 127.7, 127.5, 127.2, 127.1, 126.4, 123.7, 123.4, 121.2, 120.7, 120.6, 120.4, 120.3, 119.4, 110.4, 110.1, 110.0, 109.9, 108.8. MS (MALDI-TOF), m/z: 826.41 ([M^+^], calcd for C_61_H_38_N_4_, 826.31). Anal. Calcd for C_61_H_38_N_4_: C, 88.59; H, 4.63; N, 6.77. Found: C, 88.55; H, 4.73; N, 6.54.

## Figures and Tables

**Table 1:** Thermal, electrochemical, and photophysical data of the luminogens.

						PL *λ*_em_		*λ*_max,abs_	*Φ*_F_	*τ*_lifetime_	
	*T*_d_^a^ (°C)	*T*_g_^b^ (°C)	*E*_g_^c^ (eV)	*E*_HOMO_^d^ (eV)	*E*_LUMO_^e^ (eV)	solv^f^ (nm)	film^g^ (nm)	solv^f^ (nm)	solv^f^ (%)	solv^h^ (ns)	film^g^ (ns)
3Cz-Ph-CN	550	165	3.21	-5.59	-2.38	441	435	340	90.1	3.88	4.79
3Cz-mPh-CN	504	121	3.48	-5.58	-2.10	451	428	324	7.7	2.83	25.03
3Ph-Cz-CN	519	141	3.25	-5.62	-2.37	406	420	332	65.9	2.35	3.36

^a^5% weight loss temperature measured by TGA under N_2_. ^b^Glass transition temperature measured by DSC under N_2_. ^c^Band gap estimated from optical absorption band edge of the solution. ^d^Calculated from the onset oxidation potentials of the compounds. ^e^Estimated using empirical equations *E*_LUMO_ = *E*_HOMO_ + *E*_g_. ^f^Determined in THF solution. ^g^On glass. ^h^Observed from absorption spectra in dilute THF solution, 10 *μ*M.

**Table 2:** EL performances of 3Cz-Ph-CN (A/D/G), 3Cz-mPh-CN (B/E/H), and 3Ph-Cz-CN (C/F/I)^a^.

Device	*λ*_EL_ (nm)	*V*_on_ (V)	*L*_max_ (cd m^−2^)	*η*_P,max_ (lm W^−1^)	*η*_c,max_ (cd A^−1^)	*η*_ext,max_ (%)	CIE^b^ (*x*, *y*)
A	439	4.2	5304	1.37	2.24	2.45	0.16, 0.10
B	421	6.8	3057	0.45	1.09	1.14	0.16, 0.12
C	414 (506)	2.8	6651	24.9	23.8	18.1	0.17, 0.16
D	434	3.8	4518	1.32	1.76	2.94	0.16, 0.06
E	429	5.4	1153	0.19	0.50	0.58	0.19, 0.12
F	420	3.2	3209	1.06	1.15	1.96	0.19, 0.12
G	562	3.4	46154	59.7	80.6	26.1	0.49, 0.50
H	564	3.8	46401	56.6	77.2	25.1	0.49, 0.50
I	562	2.8	47952	84.5	84.6	27.4	0.49, 0.51
J^c^	564	2.8	48434	52.0	57.4	18.2	0.50, 0.50 [31]
K	562	3.2	47567	41.7	50.6	15.7	0.49, 0.51 [31]
L	—	2.8	—	64.5	—	24.5	0.51, 0.49 [44]
M	—	2.4	—	62.1	—	25.0	0.49, 0.49 [23]

^a^Device configuration: ITO/MoO_3_ (10 nm)/X/LiF (1 nm)/Al. For devices A-C: X = NPB (60 nm)/mCP (15 nm)/emitter (30 nm)/TPBi (30 nm). For devices D-F: X = emitter (60 nm)/TPBi (30 nm). For devices G-I: X = NPB (60 nm)/mCP (10 nm)/emitter:PO-01 (20 nm, 10 wt%)/Bphen (40 nm). ^b^Abbreviations: *V*_on_ = turn-on voltage at 1 cd m^−2^; *L*_max_ = maximum luminance; *η*_p,max_, *η*_c,max_, and *η*_ext,max_ = maximum power, current, and external quantum efficiencies, respectively; CIE = Commission International de l'Éclairage coordinates. ^c^Devices J-M: literature results for comparison.

## Supplementary Materials

Supplementary Materials Chart S1: representative blue luminogens designed by our group (note: the EQE values could not surpass 5% due to their pure fluorescent emissions). Scheme S1: detailed synthetic routes of the three luminogens. Figure S1: TGA and DSC curves of the cyano-based luminogens recorded under N_2_. Figure S2: normalized fluorescence and phosphorescence spectra of 3Cz-Ph-CN, 3Cz-mPh-CN, and 3Ph-Cz-CN at 77 K in a frozen 2-methyl-THF matrix. Concentration: 10-3 M. Figure S3: fluorescence and phosphorescence spectra of 3Cz-Ph-CN, 3Cz-mPh-CN, and 3Ph-Cz-CN at 77 K in a frozen 2-methyl-THF matrix. Concentration: 10-3 M. Figure S4: cyclic voltammograms of the three luminogens recorded in dichloromethane. Figure S5: (a) current density-voltage-luminance characteristics, (b) current efficiency-luminance characteristics, and (c) power efficiency-luminance characteristics of the nondoped devices. Device configurations: ITO/MoO_3_ (10 nm)/NPB (60 nm)/mCP (15 nm)/X (30 nm)/TPBi (30 nm)/LiF (1 nm)/Al; X refers to 3Cz-Ph-CN or 3Cz-mPh-CN or 3Ph-Cz-CN. Figure S6: EL spectra of (a) 3Cz-Ph-CN, (b) 3Cz-mPh-CN, and (c) 3Ph-Cz-CN in the nondoped devices. Device configurations: ITO/MoO_3_ (10 nm)/NPB (60 nm)/mCP (15 nm)/X (30 nm)/TPBi (30 nm)/LiF (1 nm)/Al; X refers to 3Cz-Ph-CN or 3Cz-mPh-CN or 3Ph-Cz-CN. Figure S7: transient EL spectra of 3Ph-Cz-CN in the nondoped devices. Device configurations: ITO/MoO_3_ (10 nm)/NPB (60 nm)/mCP (15 nm)/X (30 nm)/TPBi (30 nm)/LiF (1 nm)/Al; X refers to 3Ph-Cz-CN. Figure S8: current efficiency-luminance characteristics of the devices without hole-transporting layers (NPB). Inset is the diagram of energy levels. Device configurations: ITO/MoO_3_ (10 nm)/X (30 nm)/TPBi (30 nm)/LiF (1 nm)/Al. Figure S9: (a) current density-voltage-luminance characteristics, (b) external quantum efficiency-luminance characteristics, (c) current efficiency-luminance characteristics, and (d) power efficiency-luminance characteristics of the doped devices. Device configurations: ITO/MoO_3_ (10 nm)/NPB (60 nm)/mCP (10 nm)/X:PO-01 (20 nm, 10 wt%)/Bphen (40 nm)/LiF (1 nm)/Al. Figure S10: EL spectra of (a) 3Cz-Ph-CN, (b) 3Cz-mPh-CN, and (c) 3Ph-Cz-CN in the doped devices. Device configurations: ITO/MoO_3_ (10 nm)/NPB (60 nm)/mCP (10 nm)/X:PO-01 (20 nm, 10 wt%)/Bphen (40 nm)/LiF (1 nm)/Al. Figure S11: energy level diagram of the hole-only device (A), nondoped devices (B/C), and doped device (D). Figures S12–S17: ^1^H NMR and ^13^C NMR spectra of the three luminogens in deuterated chloroform. Figures S18–S20: MALDI-TOF mass spectra of the three luminogens.
