# Supplementary material for: Utilizing Electroplex Emission to Achieve External Quantum Efficiency up to 18.1% in Nondoped Blue OLED
Source: Research (Wash D C). 2020 Feb 27;2020:8649102. doi: 10.34133/2020/8649102 (PMC7063226; doi:10.34133/2020/8649102)
Supplement: Supplementary Materials — Chart S1: representative blue luminogens designed by our group (note: the EQE values could not surpass 5% due to their pure fluorescent emissions). Scheme S1: detailed synthetic routes of the three luminogens. Figure S1: TGA and DSC curves of the cyano-based luminogens recorded under N2. Figure S2: normalized fluorescence and phosphorescence spectra of 3Cz-Ph-CN, 3Cz-mPh-CN, and 3Ph-Cz-CN at 77 K in a frozen 2-methyl-THF matrix. Concentration: 10-3 M. Figure S3: fluorescence and phosphorescence spectra of 3Cz-Ph-CN, 3Cz-mPh-CN, and 3Ph-Cz-CN at 77 K in a frozen 2-methyl-THF matrix. Concentration: 10-3 M. Figure S4: cyclic voltammograms of the three luminogens recorded in dichloromethane. Figure S5: (a) current density-voltage-luminance characteristics, (b) current efficiency-luminance characteristics, and (c) power efficiency-luminance characteristics of the nondoped devices. Device configurations: ITO/MoO3 (10 nm)/NPB (60 nm)/mCP (15 nm)/X (30 nm)/TPBi (30 nm)/LiF (1 nm)/Al; X refers to 3Cz-Ph-CN or 3Cz-mPh-CN or 3Ph-Cz-CN. Figure S6: EL spectra of (a) 3Cz-Ph-CN, (b) 3Cz-mPh-CN, and (c) 3Ph-Cz-CN in the nondoped devices. Device configurations: ITO/MoO3 (10 nm)/NPB (60 nm)/mCP (15 nm)/X (30 nm)/TPBi (30 nm)/LiF (1 nm)/Al; X refers to 3Cz-Ph-CN or 3Cz-mPh-CN or 3Ph-Cz-CN. Figure S7: transient EL spectra of 3Ph-Cz-CN in the nondoped devices. Device configurations: ITO/MoO3 (10 nm)/NPB (60 nm)/mCP (15 nm)/X (30 nm)/TPBi (30 nm)/LiF (1 nm)/Al; X refers to 3Ph-Cz-CN. Figure S8: current efficiency-luminance characteristics of the devices without hole-transporting layers (NPB). Inset is the diagram of energy levels. Device configurations: ITO/MoO3 (10 nm)/X (30 nm)/TPBi (30 nm)/LiF (1 nm)/Al. Figure S9: (a) current density-voltage-luminance characteristics, (b) external quantum efficiency-luminance characteristics, (c) current efficiency-luminance characteristics, and (d) power efficiency-luminance characteristics of the doped devices. Device configurations: ITO/MoO3 (10 n [file 8649102.f1.pdf]

Supplementary Materials for

Utilizing Electroplex Emission to Achieve External Quantum Efficiency up to 18.1% in Non-doped Blue OLED

Xuejun Zhan,[1,#], Zhongbin Wu,[2,#] Yanbin Gong,[1] Jin Tu,[1] Yujun Xie,[5] Qian Peng,[4] Dongge Ma,[3,*] Qianqian Li,[1] Zhen Li[1,5,*]

[1] Department of Chemistry, Sauvage Center for Molecular Sciences, Wuhan niversity, Wuhan 430072, China

[2] Changchun Institute of Applied Chemistry, The Chinese Academy of Sciences, Changchun, 130022, China.

[3] State Key Laboratory of Luminescent Materials and Devices, South China University of Technology, Guangzhou 510640, China

[4] Institute of Chemistry, The Chinese Academy of Sciences, Beijing 100190, China

[5] Institute of Molecular Aggregation Science, Tianjin University, Tianjin 300072, China

* Correspondence should be addressed to Zhen Li; lizhen@whu.edu.cn or lichemlab@163.com and Dongge Ma; msdgma@scut.edu.cn

[#] Dr. X. Zhan and Dr. Z. Wu contributed equally to this work.

This file includes:

Chart S1. Representative blue luminogens designed by our group (note: the EQE values couldn't surpass 5% due to their pure fluorescent emissions).[1-6]

Scheme S1. Detailed synthetic routes of the three luminogens.

Figure S1. TGA and DSC curves of the cyano-based luminogens recorded under N_2.

Figure S2. Normalized fluorescence and phosphorescence spectra of 3Cz-Ph-CN, 3Cz-mPh-CN and 3Ph-Cz-CN at 77 K in a frozen 2-methyl-THF matrix. Concentration: 10^{-3} M.

Figure S3. Fluorescence and phosphorescence spectra of 3Cz-Ph-CN, 3Cz-mPh-CN and 3Ph-Cz-CN at 77 K in a frozen 2-methyl-THF matrix. Concentration: 10^{-3} M.

Figure S4. Cyclic voltammograms of the three luminogens recorded in dichloromethane.

Figure S5. (a) Current density-voltage-luminance characteristics, (b) current efficiency-luminance characteristics, and (c) power efficiency-luminance characteristics of the non-

doped devices. Device configurations: ITO/MoO_3 (10 nm)/NPB (60 nm)/mCP (15 nm)/X (30 nm)/TPBi (30 nm)/LiF (1 nm)/Al, X refers to 3Cz-Ph-CN or 3Cz-mPh-CN or 3Ph-Cz-CN.

Figure S6. EL spectra of (a) 3Cz-Ph-CN, (b) 3Cz-mPh-CN, and (c) 3Ph-Cz-CN in the non-doped devices. Device configurations: ITO/MoO_3 (10 nm)/NPB (60 nm)/mCP (15 nm)/X (30 nm)/TPBi (30 nm)/LiF (1 nm)/Al, X refers to 3Cz-Ph-CN or 3Cz-mPh-CN or 3Ph-Cz-CN.

Figure S7. Transient EL spectra of 3Ph-Cz-CN in the non-doped devices. Device configurations: ITO/MoO_3 (10 nm)/NPB (60 nm)/mCP (15 nm)/X (30 nm)/TPBi (30 nm)/LiF (1 nm)/Al, X refers to 3Ph-Cz-CN.

Figure S8. Current efficiency-luminance characteristics of the devices without hole-transporting layers (NPB). Inset is the diagram of energy levels. Device configurations: ITO/MoO_3 (10 nm)/X (30 nm)/TPBi (30 nm)/LiF (1 nm)/Al.

Figure S9. (a) Current density-voltage-luminance characteristics, (b) external quantum efficiency-luminance characteristics, (c) current efficiency-luminance characteristics, and (d) power efficiency-luminance characteristics of the doped devices. Device configurations: ITO/MoO_3 (10 nm)/NPB (60 nm)/mCP (10 nm)/X:PO-01 (20 nm, 10 wt%)/Bphen (40 nm)/LiF (1 nm)/Al.

Figure S10. EL spectra of (a) 3Cz-Ph-CN, (b) 3Cz-mPh-CN, and (c) 3Ph-Cz-CN in the doped devices. Device configurations: ITO/MoO_3 (10 nm)/NPB (60 nm)/mCP (10 nm)/X:PO-01 (20 nm, 10 wt%)/Bphen (40 nm)/LiF (1 nm)/Al.

Figure S11. Energy level diagram of the hole-only device (A), non-doped devices (B/C) and doped device (D).

Figure S12-17. ^{1}H NMR and ^{13}C NMR spectra of the three luminogens in deuterated chloroform.

Figure S18-20. MALDI-TOF mass spectra of the three luminogens.

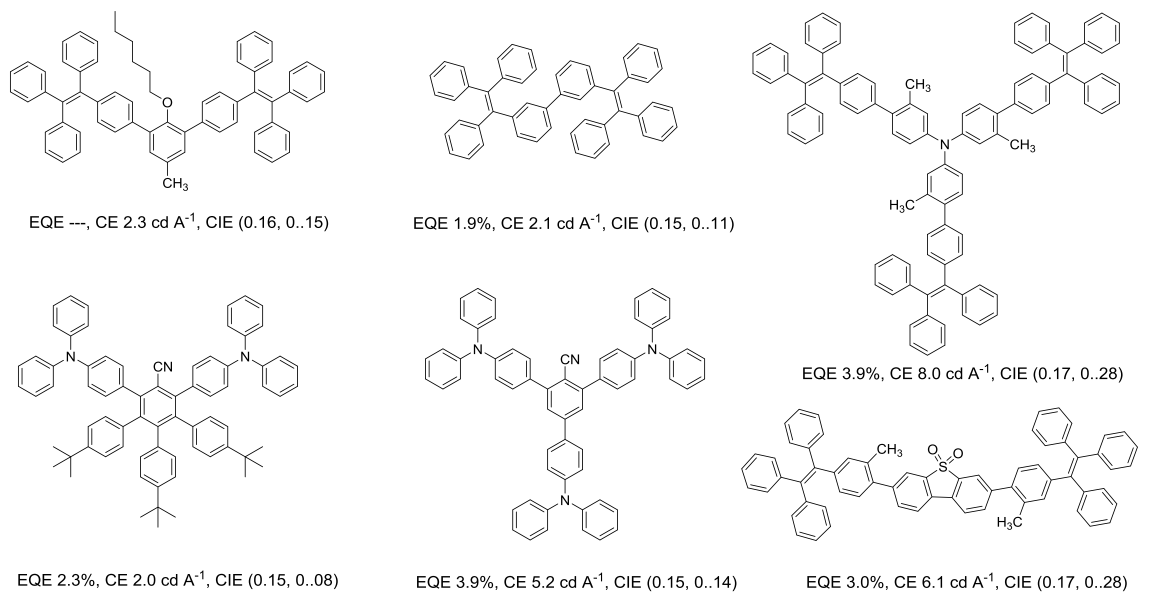

Chart S1. Representative blue luminogens designed by our group (note: the EQE values couldn't surpass 5% due to their pure fluorescent emissions).[1-6]

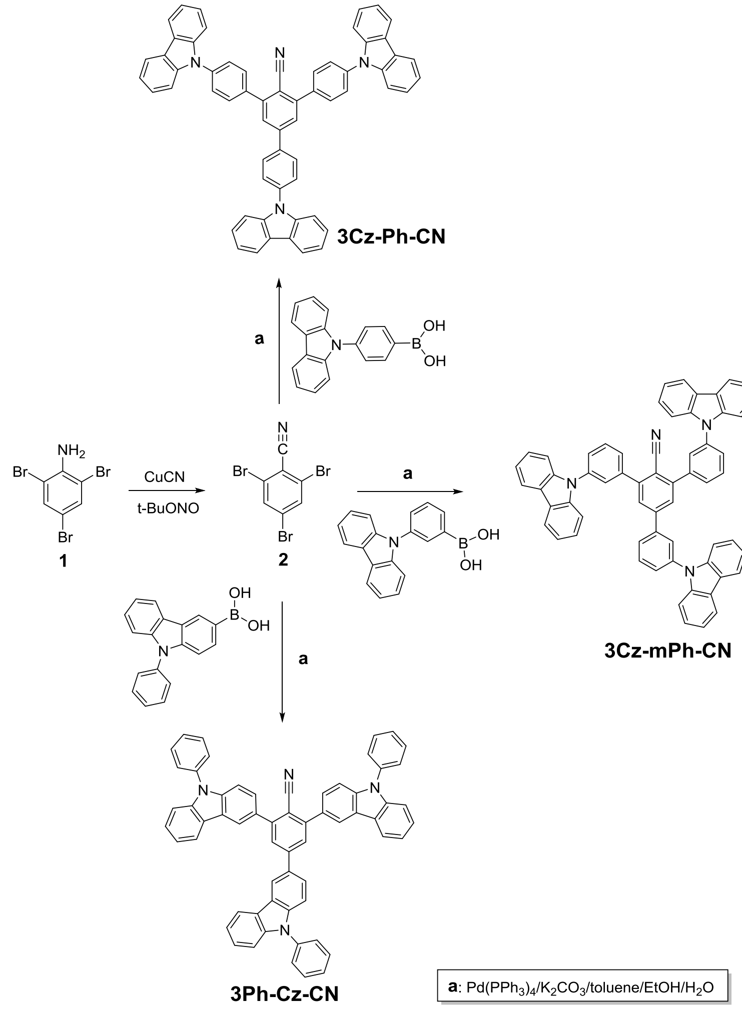

Scheme S1. Detailed synthetic routes of the three luminogens.

Experimental Section

Characterization: ^{1}H and ^{13}C NMR spectra were measured on WNMR-I NMR (600 MHz) and Bruker AVANCE III HD (400 MHz) spectrometers, respectively. Elemental analyses of carbon (C), hydrogen (H), and nitrogen (N) were performed on a CARLOERBA-1106 microanalyzer. MS (EI) spectrum was recorded on a Finnigan PRACE mass spectrometer. MALDI-TOF spectra were measured with Bruker Autoflex III mass spectrometer operating in MALDI-TOF (matrix-assisted laserdesorption/ionization-time-of-flight) mode with 1,8,9-anthracenetriol as the matrix. For photophysical properties: UV-vis absorption spectra were recorded on a Shimadzu UV-2500 recording spectrometer while the photoluminescence spectra were recorded on a Hitachi F-4500 fluorescence spectrometer. For low temperature tests, samples were all cooled in liquid nitrogen. The emission decay profiles were measured with FLS980 fluorescence lifetime spectrometer. In the atmosphere of nitrogen, thermogravimetric analysis (TGA) and differential scanning calorimetry (DSC) were performed with NETZSCH STA 449C and Mettler Toledo DSC 822e instrument, respectively. Cyclic voltammetry (CV) curves of the three emitters were obtained on a CHI voltammetric analyzer in a three-electrode cell. The three electrodes were Pt counter electrode, Ag/AgCl reference electrode, and glassy carbon working electrode. The scans were performed at the rate of 100 mV s^{-1} with tetrabutylammonium perchlorate (0.1 M, anhydrous dichloromethane solution purged with nitrogen) as the supporting electrolyte. All the potential values obtained were converted to values versus the saturated calomel electrode (SCE) by using ferrocenium/ferrocene (Fc^{+}/Fc) as internal standard. The electronic and geometrical properties of all the three luminogens were optimized by Gaussian 09 program (B3LYP/6-31g(d) level).

Synthesis of the compounds:

2,4,6-Tribromoaniline (**1**) and the three boric acids of (4-(9*H*-carbozol-9-yl)phenyl)boronic acid\(3-(9*H*-carbozol-9-yl)phenyl)boronic acid\(9-phenyl-9*H*-carbazol-3-yl)boronic acid were

commercial available. All other chemicals and reagents were obtained from commercial sources and used as received without further purification. Solvents for chemical synthesis were purified according to the standard procedures.

*Synthesis of 2,4,6-tribromobenzonitrile (**2**)*: A mixture of 2,4,6-tribromoaniline (**1**, 327 mg, 1.0 mmol) and CuCN (135 mg, 1.5 mmol) were added to anhydrous DMSO (40 mL) at the temperature of 60 ^{o}C. After string for 30 minutes, *tert*-butyl nitrite (0.357 mL, 3.0 mmol) was slowly added. The resultant mixture was allowed to stir for 4 h, then poured into 1 M HCl (100 mL) and extracted with chloroform. The combined organic extracts were dried over anhydrous Na_2SO_4 and concentrated by rotary evaporation. The crude product was purified by column chromatography on silica gel using chloroform/petroleum ether as eluent to give a pale yellow solid in the yield of 27% (91 mg). ^{1}H NMR (600 MHz, $CDCl_3$) δ (ppm): 7.61 (s, 2H). ^{13}C NMR (100 MHz, $CDCl_3$) δ (ppm): 134.6, 128.1, 127.0, 117.8, 115.4. MS (EI), m/z: 338.49 ([M$^+$], calcd for $C_7H_2Br_3N$, 336.77). Anal. Calcd for $C_7H_2Br_3N$: C, 24.74; H, 0.59; N, 4.12. Found: C, 24.99; H, 0.73; N, 4.27.

Synthetic procedures of **3Cz-Ph-CN**, **3Cz-mPh-CN** and **3Ph-Cz-CN** could be found in experimental section of the main manuscript. NMR spectra and MALDI-TOF mass spectra were shown in Figure S11-S19.

References:

1 Huang, J. et al. Benzene-cored fluorophors with TPE peripheries: facile synthesis, crystallization-induced blue-shifted emission, and efficient blue luminogens for non-doped OLEDs. Journal of Materials Chemistry vol. 22, no. 24, pp. 12001-12007, 2012.

2 Huang, J. et al. Similar or totally different: the control of conjugation degree through minor structural modifications, and deep-blue aggregation induced emission luminogens for non-doped OLEDs. *Advanced Functional Materials*, vol. 23, no. 18, pp. 2329-2337, 2013.

3 Huang, J. et al. Blue aggregation induced emission luminogens: high external quantum efficiencies up to 3.99% in LED device, and restriction of the conjugation length through

rational molecular design. *Advanced Functional Materials*, vol. 24, no. 48, pp. 7645-7654, 2014.

4 Zhan, X. et al. New AIEgens containing dibenzothiophene-S,S-dioxide and tetraphenylethene moieties: similar structures but very different hole/electron transport properties. *Journal of Materials Chemistry C* , vol. 3, no. 23, pp. 5903-5909, 2015.

5 Zhan, X. et al. Polyphenylbenzene as a platform for deep-blue OLEDs: aggregation enhanced emission and high external quantum efficiency of 3.98%. *Chemistry of Materials*, vol. 27, no. 5, pp. 1847-1854, 2015.

6 Zhan, X. et al. Benzene-cored AIEgens for deep-blue OLEDs: high performance without hole-transporting layers, and unexpected excellent host for orange emission as a side-effect. *Chemical Science*, vol. 7, no. 7, pp. 4355-4363, 2016.

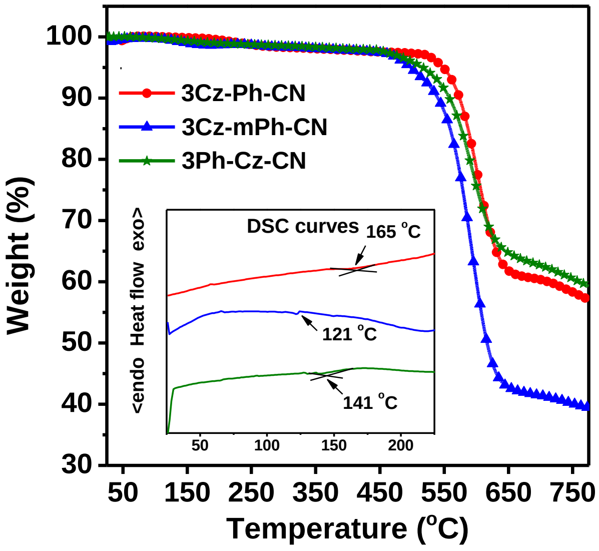

Figure S1. TGA and DSC curves of the cyano-based luminogens recorded under N_2.

 6

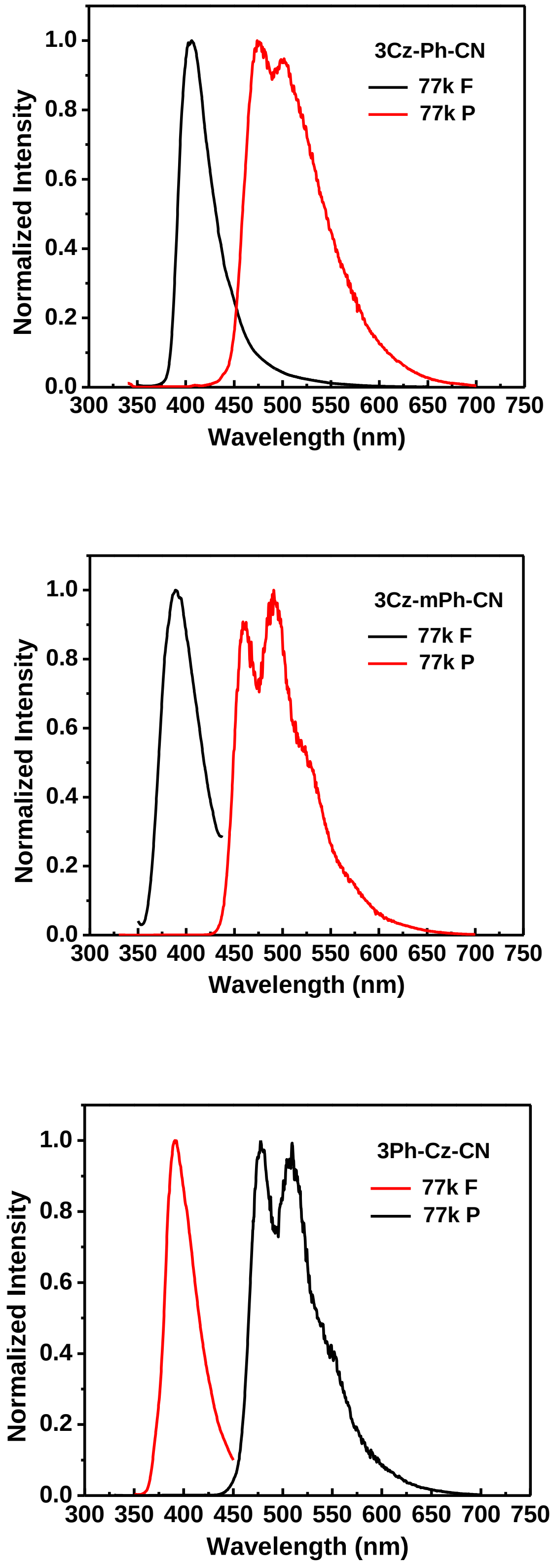

Figure S2. Normalized fluorescence and phosphorescence spectra of 3Cz-Ph-CN, 3Cz-mPh-CN and 3Ph-Cz-CN at 77 K in a frozen 2-methyl-THF matrix. Concentration: 10^{-3} M.

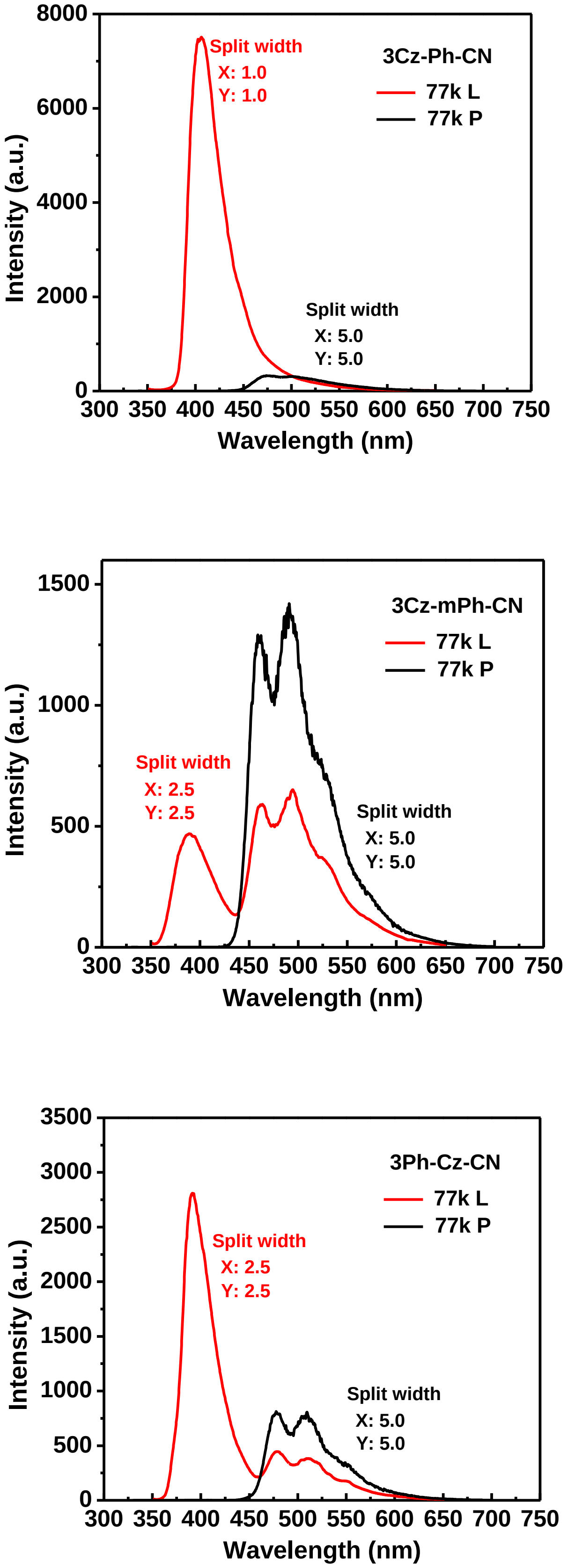

Figure S3. Fluorescence and phosphorescence spectra of 3Cz-Ph-CN, 3Cz-mPh-CN and 3Ph-Cz-CN at 77 K in a frozen 2-methyl-THF matrix. Concentration: 10^{-3} M.

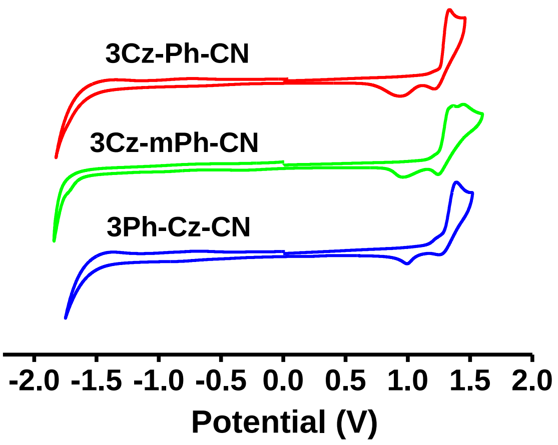

Figure S4. Cyclic voltammograms of the three luminogens recorded in dichloromethane. The three electrodes were Pt counter electrode, Ag/AgCl reference electrode, and glassy carbon working electrode. The scans were performed at the rate of 100 mV s^{-1} with tetrabutylammonium perchlorate (0.1 M, anhydrous dichloromethane solution purged with nitrogen) as the supporting electrolyte. All the potential values obtained were converted to values versus the saturated calomel electrode (SCE) by using ferrocenium/ferrocene (Fc^{+}/Fc) as internal standard.

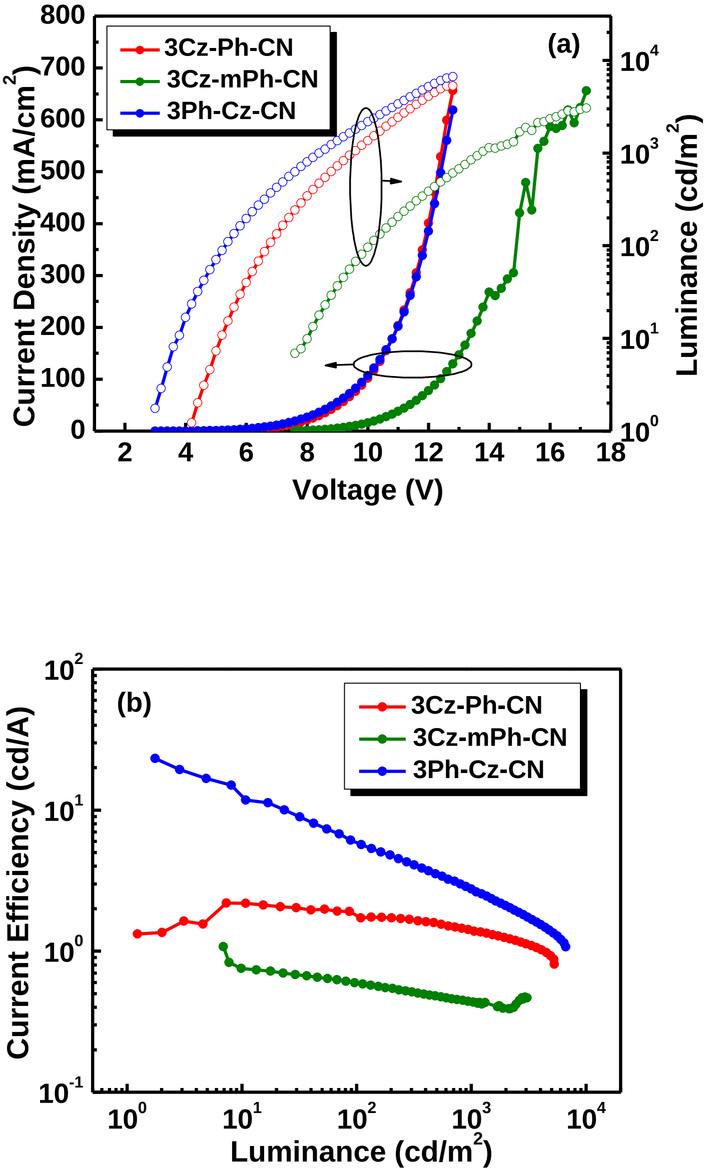
(a)
3Cz-Ph-CN
3Cz-mPh-CN
3Ph-Cz-CN
Current Density (mA/cm^2)
Luminance (cd/m^2)
Voltage (V)
(b)
3Cz-Ph-CN
3Cz-mPh-CN
3Ph-Cz-CN
Current Efficiency (cd/A)
Luminance (cd/m^2)

 10

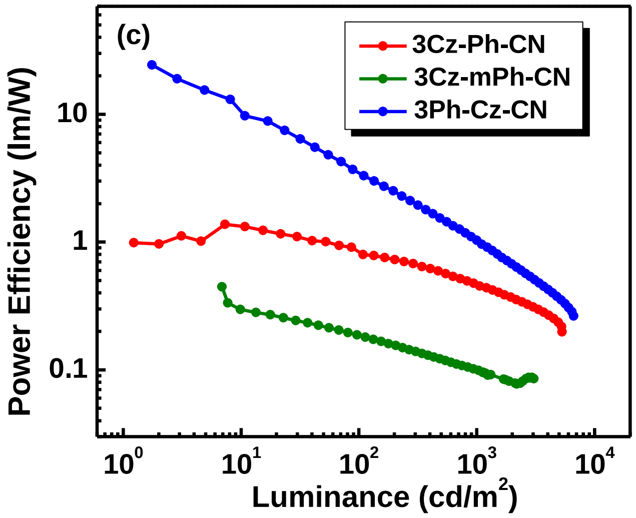

Figure S5. (a) Current density-voltage-luminance characteristics, (b) current efficiency-luminance characteristics, and (c) power efficiency-luminance characteristics of the non-doped devices. Device configurations: ITO/MoO_3 (10 nm)/NPB (60 nm)/mCP (15 nm)/X (30 nm)/TPBi(30 nm)/LiF (1 nm)/Al, X refers to 3Cz-Ph-CN or 3Cz-mPh-CN or 3Ph-Cz-CN.

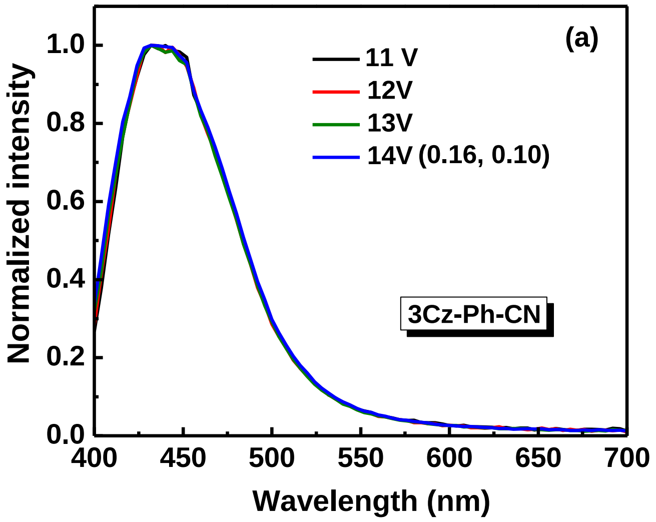

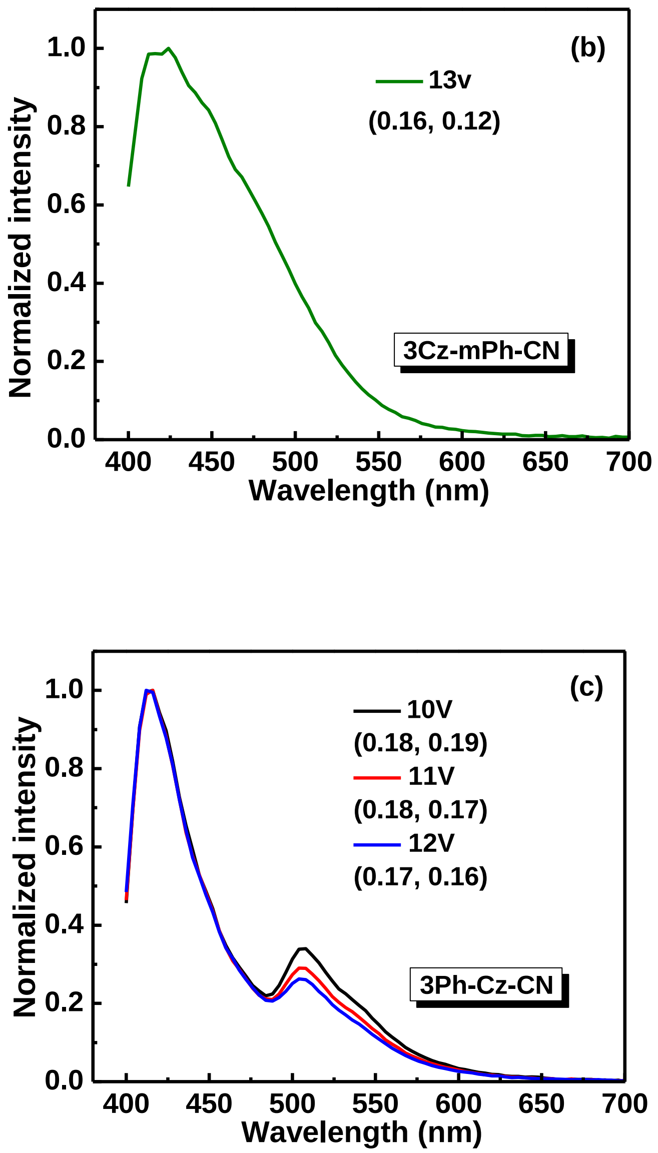

Figure S6. EL spectra of (a) 3Cz-Ph-CN, (b) 3Cz-mPh-CN, and (c) 3Ph-Cz-CN in the non-doped devices. Device configurations: ITO/MoO_3 (10 nm)/NPB (60 nm)/mCP (15 nm)/X (30 nm)/TPBi (30 nm)/LiF (1 nm)/Al, X refers to 3Cz-Ph-CN or 3Cz-mPh-CN or 3Ph-Cz-CN.

 12

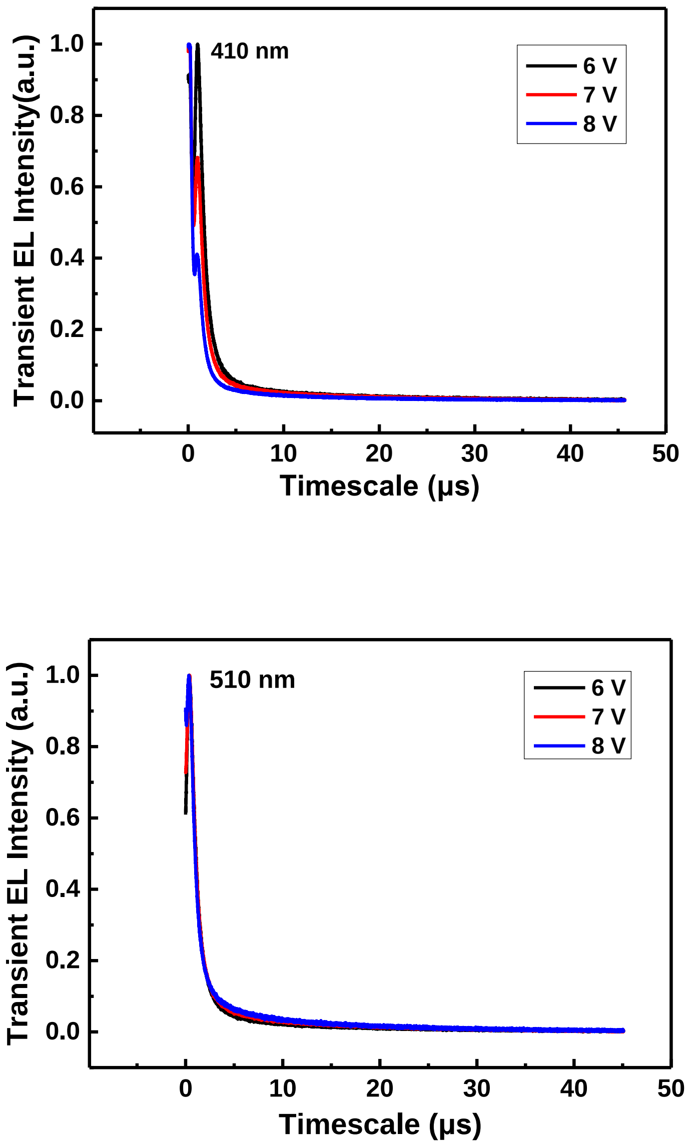

Figure S7. Transient EL spectra of 3Ph-Cz-CN in the non-doped devices. Device configurations: ITO/MoO_3 (10 nm)/NPB (60 nm)/mCP (15 nm)/X (30 nm)/TPBi (30 nm)/LiF (1 nm)/Al, X refers to 3Ph-Cz-CN.

13

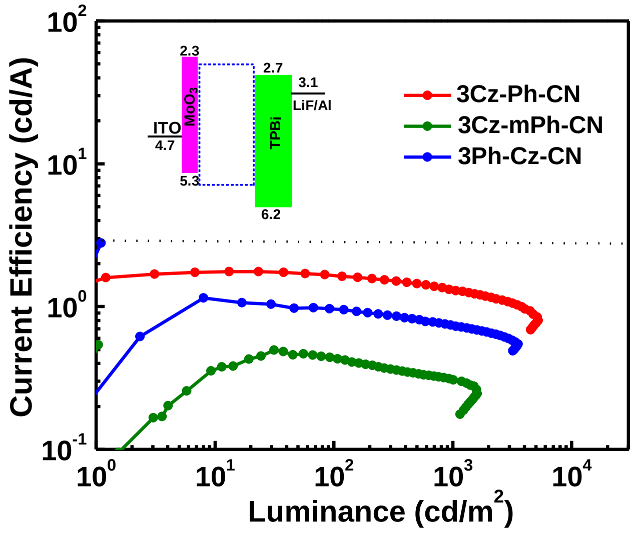

Figure S8. Current efficiency-luminance characteristics of the devices without hole-transporting layers (NPB). Insert is the diagram of energy levels. Device configurations: ITO/MoO_3 (10 nm)/X (30 nm)/TPBi (30 nm)/LiF (1 nm)/Al.

14

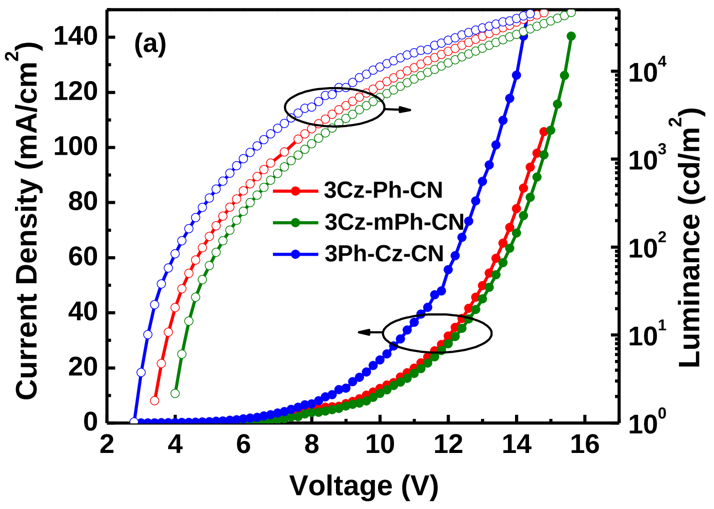
(a)
Current Density (mA/cm^2)
Luminance (cd/m^2)
Voltage (V)
3Cz-Ph-CN
3Cz-mPh-CN
3Ph-Cz-CN
0
20
40
60
80
100
120
140
2
4
6
8
10
12
14
16
10^0
10^1
10^2
10^3
10^4

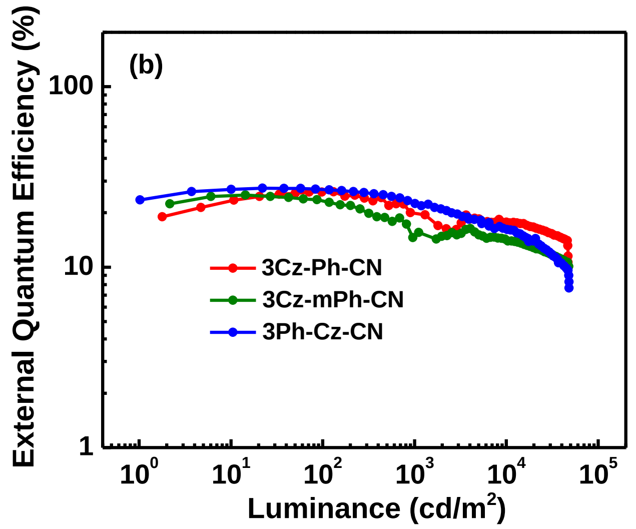
(b)
External Quantum Efficiency (%)
Luminance (cd/m^2)
3Cz-Ph-CN
3Cz-mPh-CN
3Ph-Cz-CN
1
10
100
10^0
10^1
10^2
10^3
10^4
10^5

15

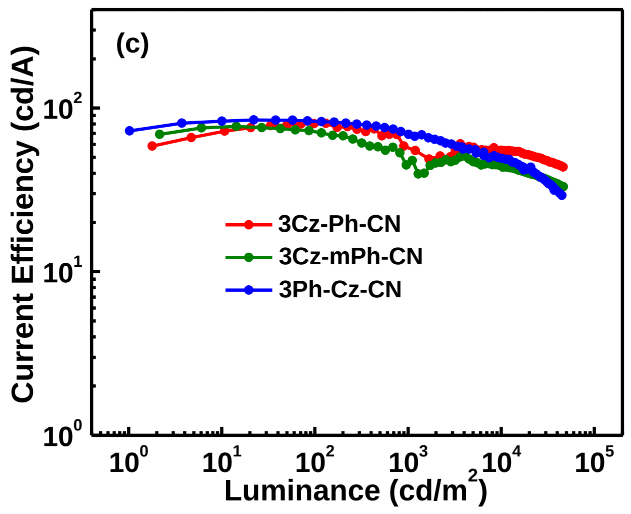

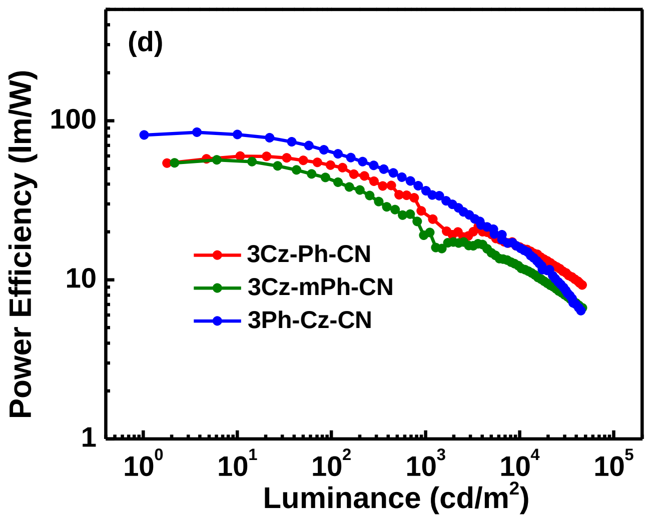

Figure S9. (a) Current density-voltage-luminance characteristics, (b) external quantum efficiency-luminance characteristics, (c) current efficiency-luminance characteristics, and (d) power efficiency-luminance characteristics of the doped devices. Device configurations: ITO/MoO_3 (10 nm)/NPB (60 nm)/mCP (10 nm)/X:PO-01 (20 nm, 10 wt%)/Bphen (40 nm)/LiF (1 nm)/Al.

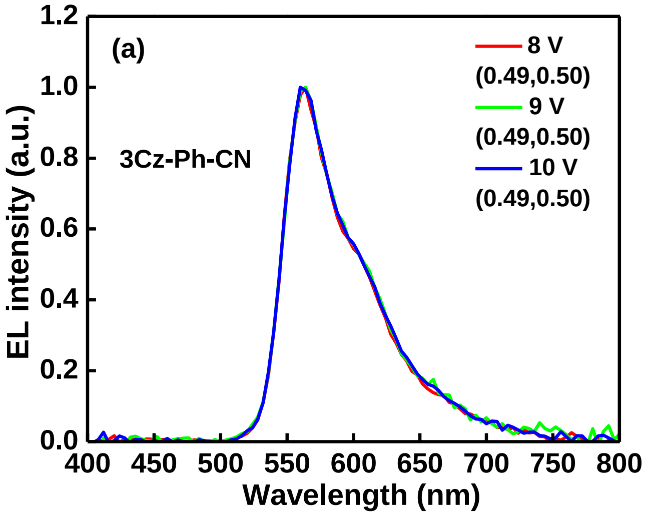
(a)
3Cz-Ph-CN
8 V
(0.49,0.50)
9 V
(0.49,0.50)
10 V
(0.49,0.50)
EL intensity (a.u.)
Wavelength (nm)
1.2
1.0
0.8
0.6
0.4
0.2
0.0
400 450 500 550 600 650 700 750 800

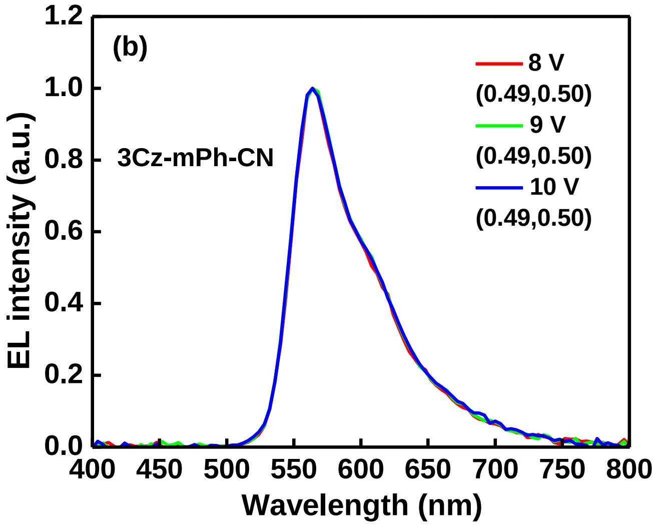
(b)
3Cz-mPh-CN
8 V
(0.49,0.50)
9 V
(0.49,0.50)
10 V
(0.49,0.50)
EL intensity (a.u.)
Wavelength (nm)
1.2
1.0
0.8
0.6
0.4
0.2
0.0
400 450 500 550 600 650 700 750 800

 17

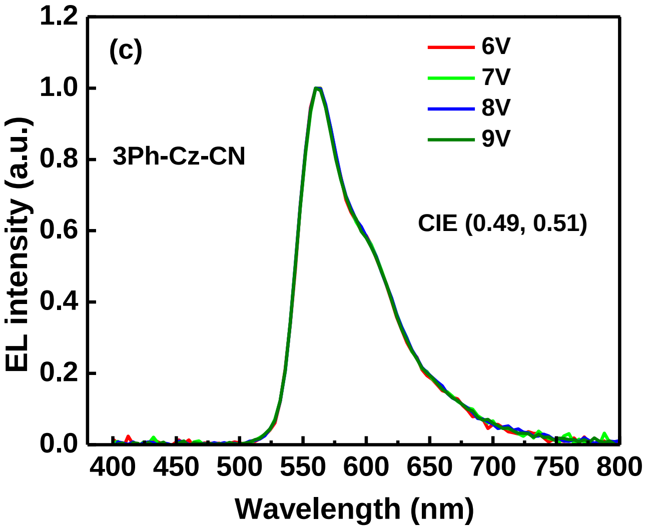

Figure S10. EL spectra of (a) 3Cz-Ph-CN, (b) 3Cz-mPh-CN, and (c) 3Ph-Cz-CN in the doped devices. Device configurations: ITO/MoO_3 (10 nm)/NPB (60 nm)/mCP (10 nm)/X:PO-01 (20 nm, 10 wt%)/Bphen (40 nm)/LiF (1 nm)/Al.

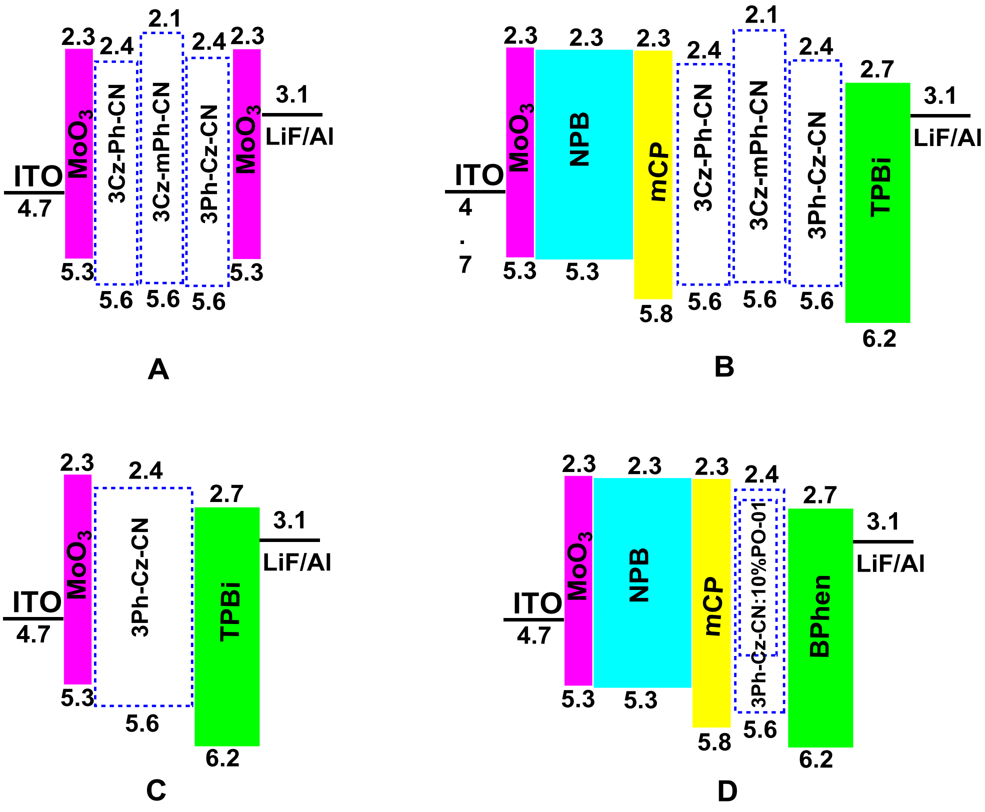

Figure S11. Energy level diagram of the hole-only device (A), non-doped devices (B/C) and doped device (D).

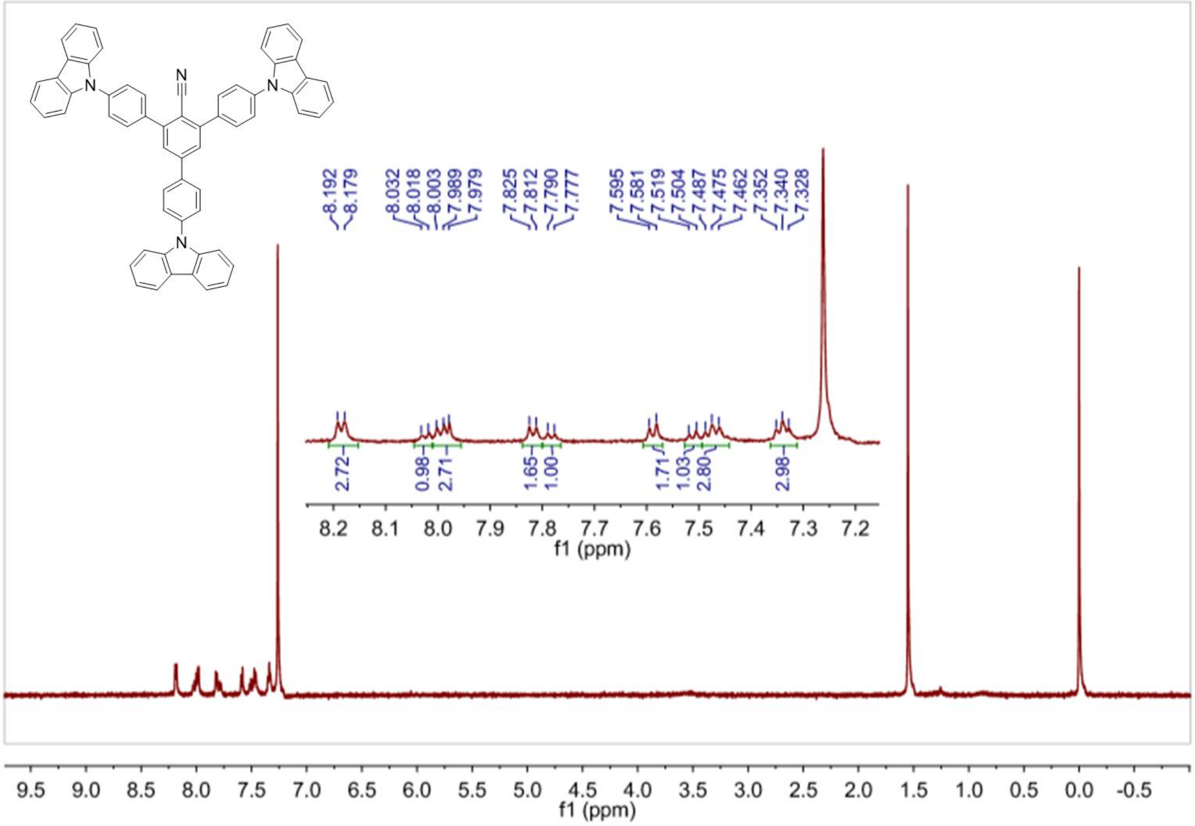

Figure S12. ^{1}H NMR spectrum of 3Cz-Ph-CN in deuterated chloroform.

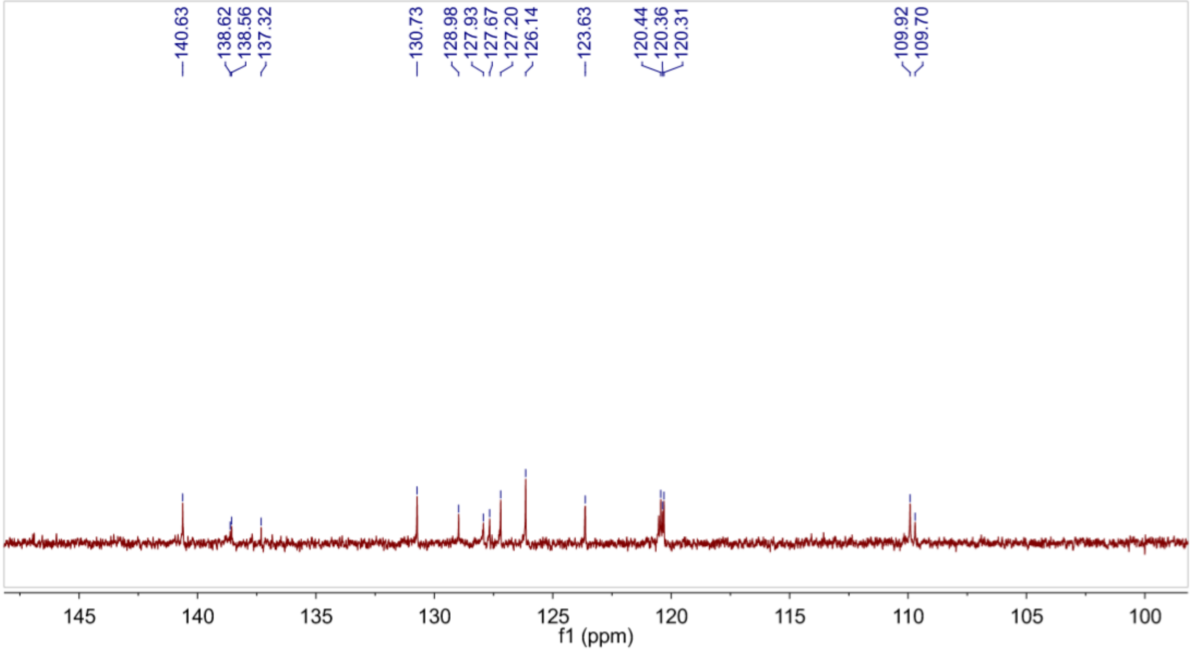

Figure S13. ^{13}C NMR spectrum of 3Cz-Ph-CN in deuterated chloroform.

19

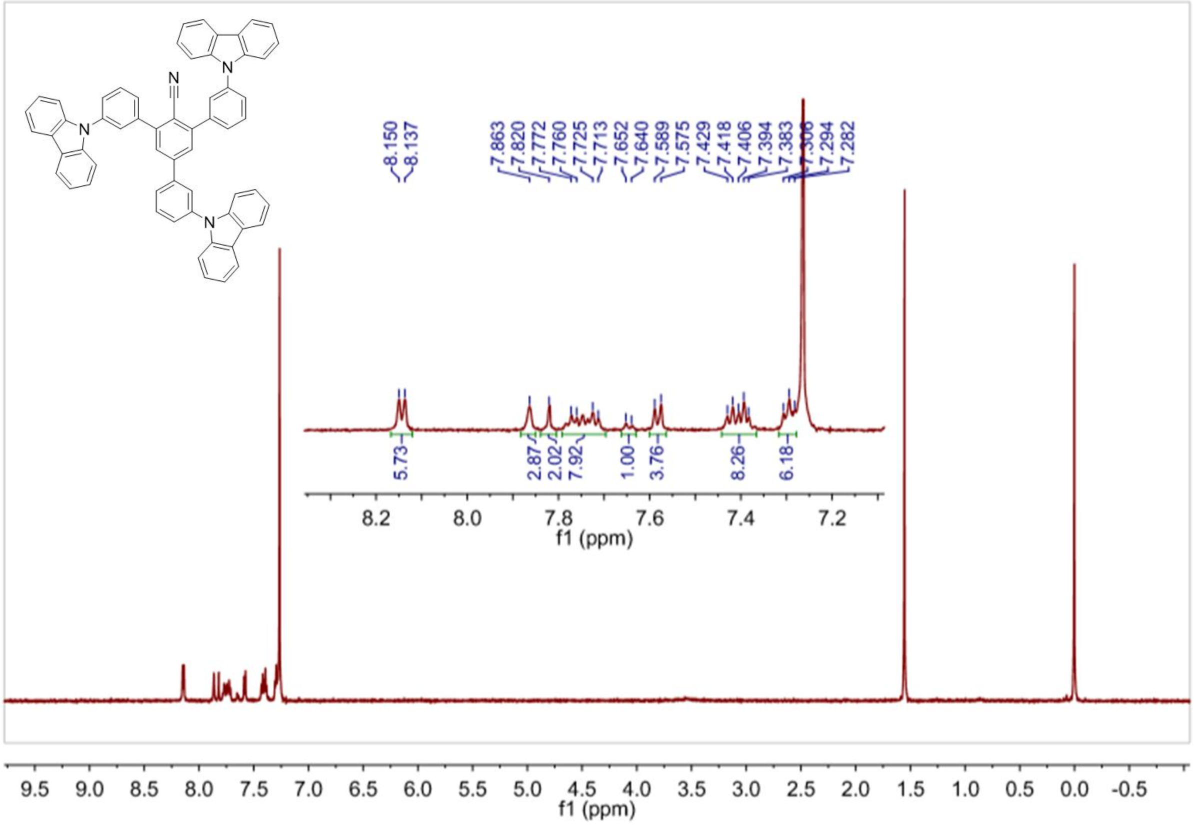

Figure S14. ^{1}H NMR spectrum of 3Cz-mPh-CN in deuterated chloroform.

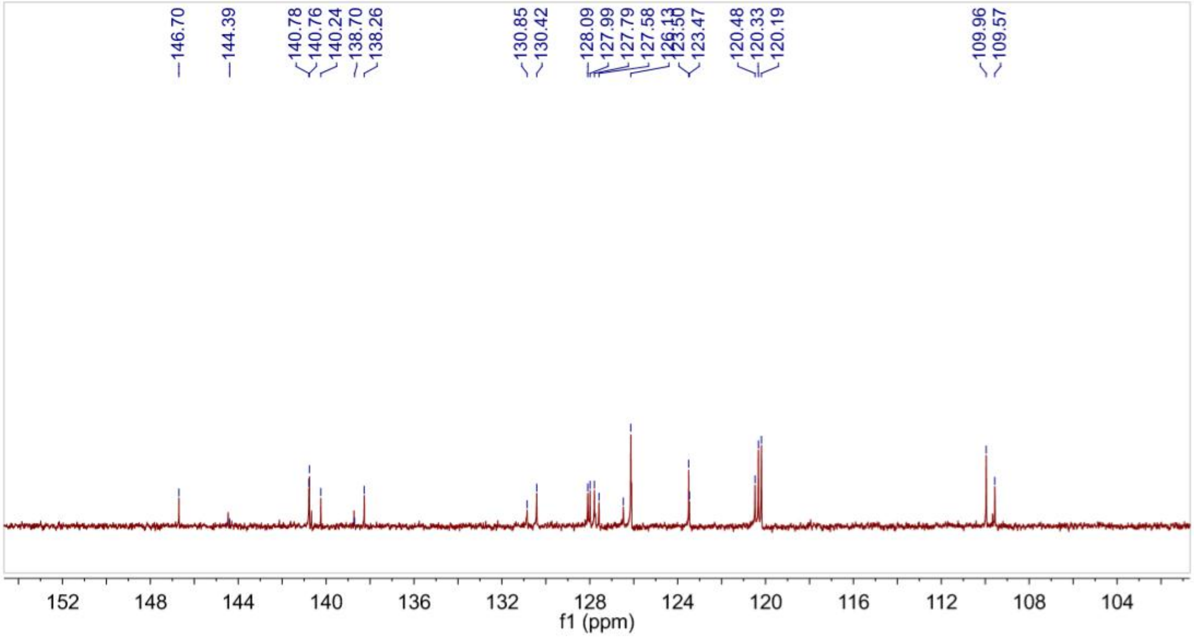

Figure S15. ^{13}C NMR spectrum of 3Cz-mPh-CN in deuterated chloroform.

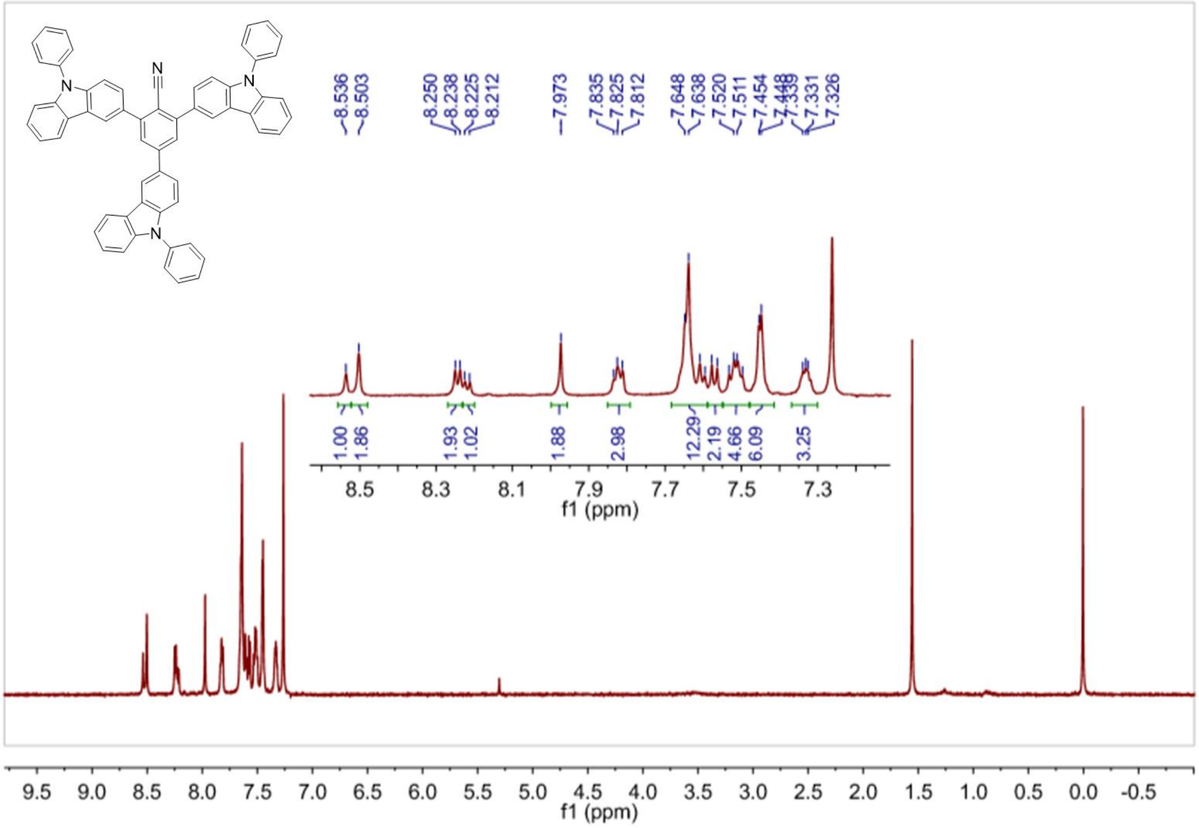

Figure S16. ^{1}H NMR spectrum of 3Ph-Cz-CN in deuterated chloroform.

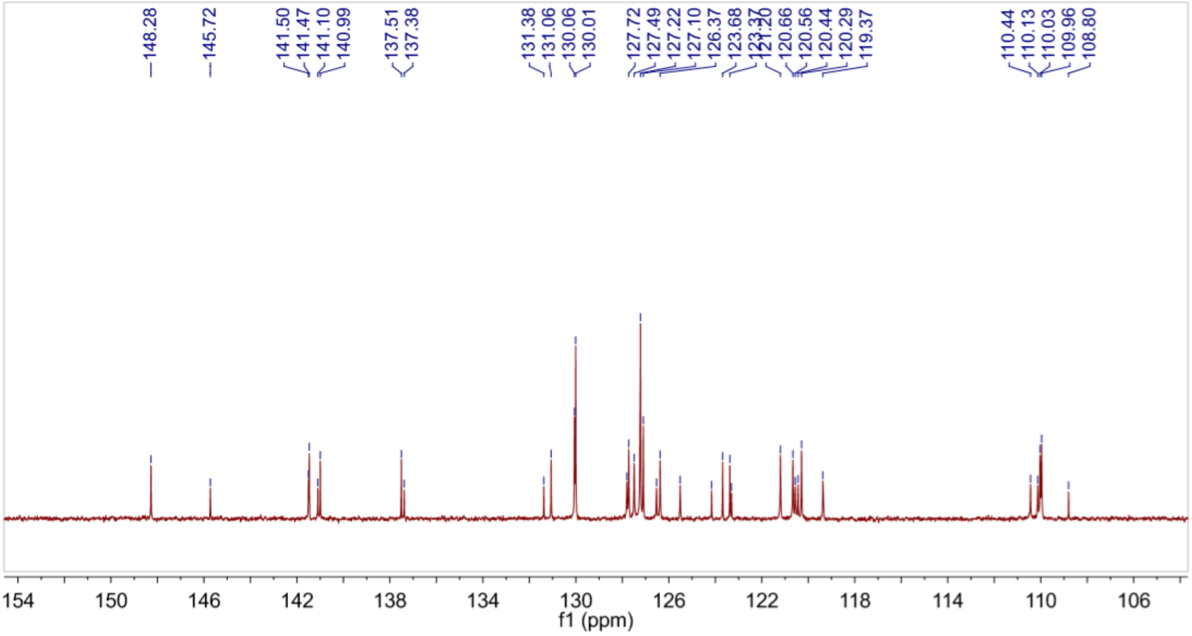

Figure S17. ^{13}C NMR spectrum of 3Ph-Cz-CN in deuterated chloroform.

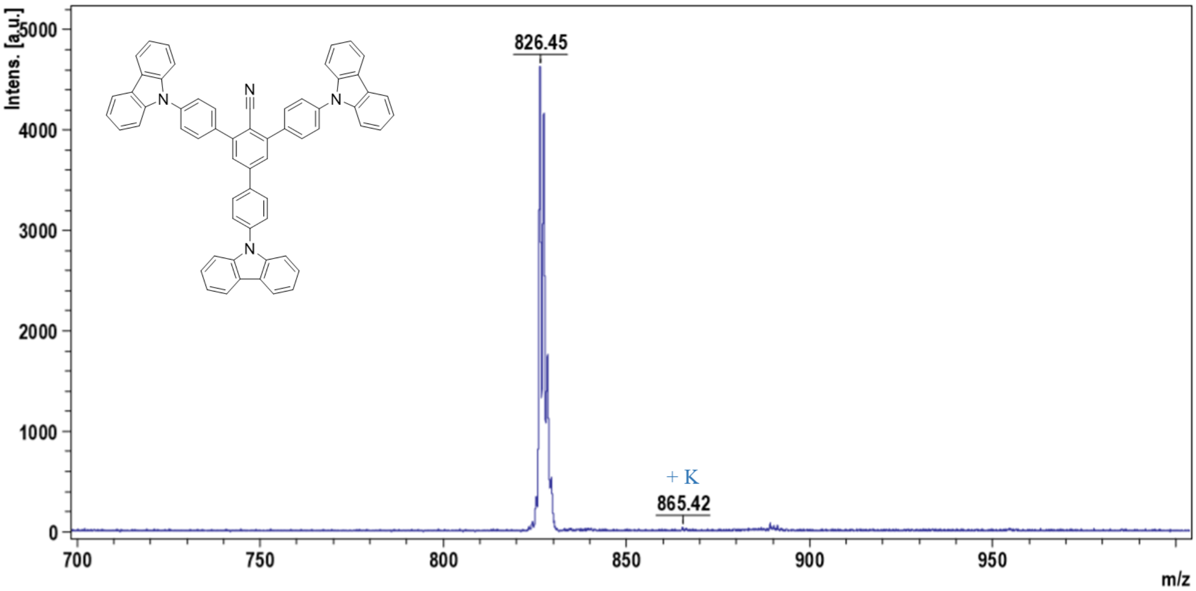

Figure S18. MALDI-TOF spectrum of 3Cz-Ph-CN.

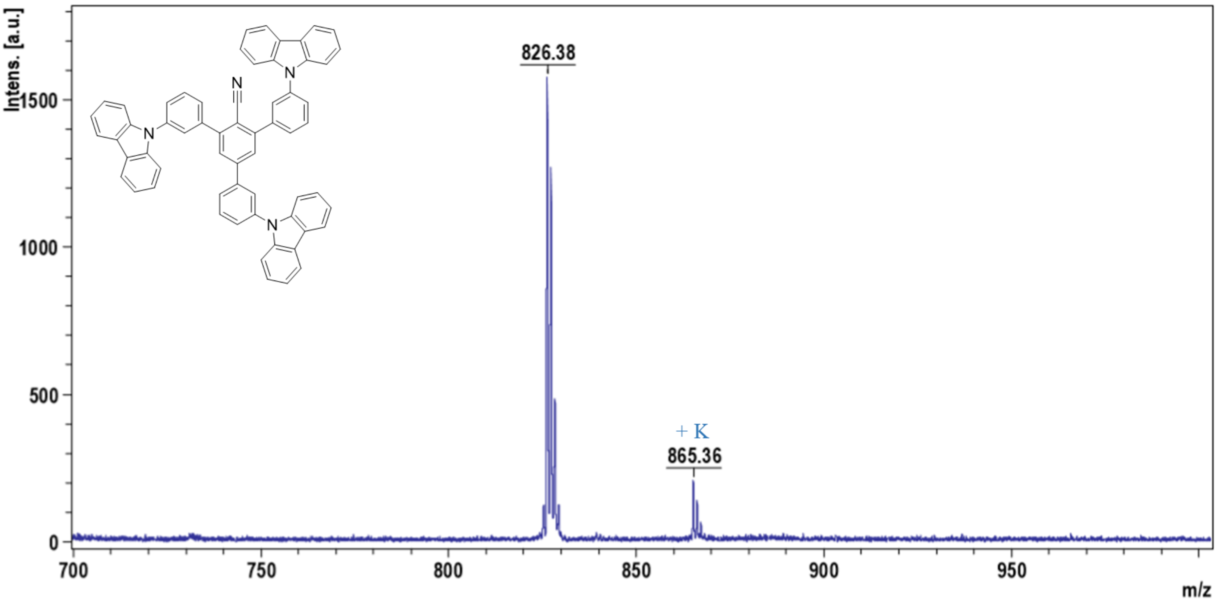

Figure S19. MALDI-TOF spectrum of 3Cz-mPh-CN.

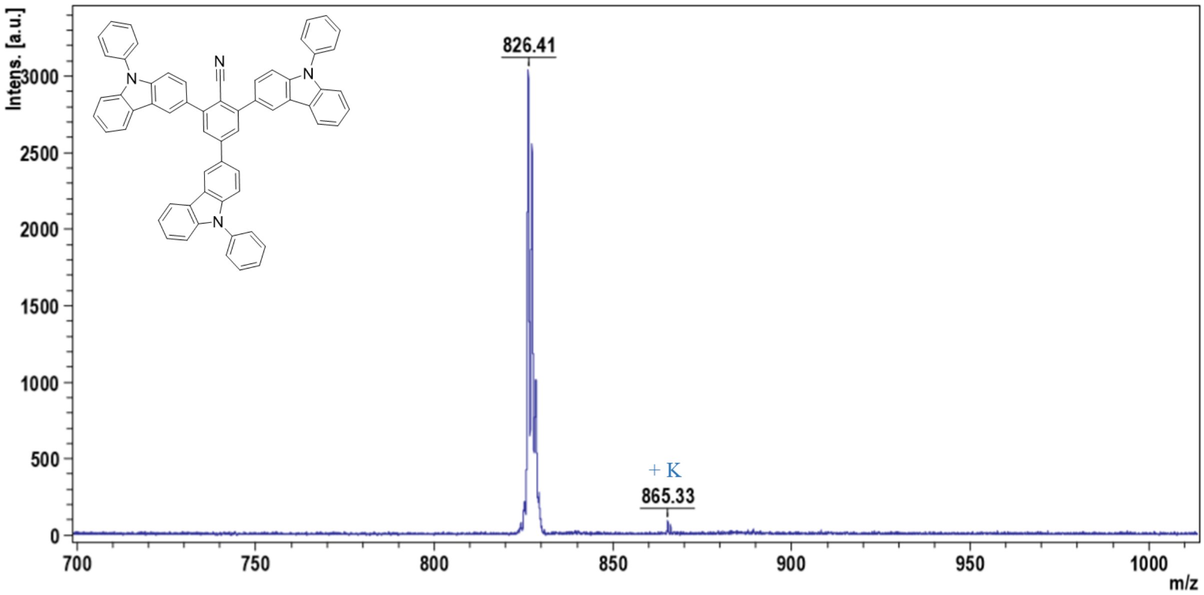

Figure S20. MALDI-TOF spectrum of 3Ph-Cz-CN.

23
